# Differential decreases in various HIV DNA regions and HIV transcripts after ART initiation during chronic infection

**DOI:** 10.1128/jvi.00683-25

**Published:** 2025-07-08

**Authors:** Julie Janssens, Cordelia Isbell, Sun Jin Kim, Adam Wedrychowski, Alton Barbehenn, Rebecca Hoh, Satish K. Pillai, Michael J. Peluso, Sulggi A. Lee, Timothy J. Henrich, Nadia R. Roan, Steven G. Deeks, Steven A. Yukl

**Affiliations:** 1 Department of Medicine, University of California, San Francisco (UCSF) 8785, San Francisco, California, USA; 2 Vitalant Research Institute 166672 , San Francisco, California, USA; 3 Department of Urology, University of California, San Francisco (UCSF) 8785, San Francisco, California, USA; 4 Gladstone Institutes 40292, San Francisco, California, USA; The Ohio State University, Columbus, Ohio, USA

**Keywords:** HIV-1, HIV, ART, transcription, RNA, DNA

## Abstract

HIV-transcribing cells persist on ART and likely contribute to inflammation as well as the viral rebound that occurs after stopping ART. However, it is unclear how ART start time and prolonged time on ART affect the clearance of cells expressing HIV transcripts that differ in their processivity and/or the presence of mutations. To investigate these questions, we measured levels of various HIV transcripts and their corresponding HIV DNA regions in longitudinal samples of peripheral CD4+ T cells obtained from 10 individuals during untreated chronic infection (T1) and up to four additional timepoints on suppressive ART (median years after ART start: T2 = 0.9; T3 = 2.9; T4 = 5.2; T5 = 7). Before ART, the pattern of HIV transcripts suggested blocks to elongation and completion, and only ~1% of intact proviruses were transcribing intact HIV RNA. After ART, we observed decreases in most HIV transcripts. Multiply spliced HIV RNA tended to decrease faster than initiated HIV RNA, and intact HIV RNA decreased faster than 5′ defective HIV RNA. ART reduced completed but not elongated or Pol HIV RNA as normalized to HIV DNA. While most proviruses and HIV transcripts showed no further decline after T2, multiply spliced HIV RNA continued to decline through T3, and R-U5-pre-Gag HIV DNA continued to decline through T4, suggesting their clearance may be dictated by different or more sustained immune mechanisms. No further changes were observed after T4. These findings suggest the need for new therapies that can target the remaining proviruses/transcripts and/or modulate the expression of completed, multiply spliced, and intact HIV RNA.

**IMPORTANCE:**

Even in ART-treated people living with HIV (PWH), expression of different viral products may contribute to immune activation, inflammation, organ damage, and reduced life expectancy. We quantified different types of HIV DNA and transcripts in people living with untreated chronic HIV and up to 4 timepoints over 6–10 years on ART. During the first year on ART, multiply spliced HIV RNA decreased faster than total HIV RNA, and intact HIV RNA decreased faster than defective RNA. Multiply spliced HIV RNA continued to decline over 1–3 years on ART, and proviruses containing the 5′ end declined over years 3–5, suggesting differences in the immune clearance of various HIV transcripts and proviruses. While intact HIV RNA became undetectable, levels of incomplete or defective HIV transcripts reached an equilibrium after 3 years on ART, indicating the limits of the immune system and ART to reduce incomplete/defective HIV transcripts that may still contribute to immune activation.

## INTRODUCTION

Although combination antiretroviral therapy (ART) can suppress the replication of HIV and reduce morbidity and mortality, it does not fully restore health or cure HIV. HIV persists in various subsets of infected cells, including a large number of cells with proviral DNA that is not infectious (defective) because of mutations such as deletions and hypermutations (1), a much smaller number with intact proviruses (2), and an even smaller fraction that can be induced by activation to produce infectious virions (the latent reservoir) (3–5). These latently infected cells may overlap with the “rebound competent” reservoir that is responsible for the rebound of plasma virus after stopping ART.

While most research has focused on the intact and latent reservoirs, there is also an “active” reservoir of infected cells that spontaneously transcribe HIV RNA *in vivo*. Since ART does not prevent HIV transcription from cells that were already infected, subsets of both defective and intact proviruses may express various forms of HIV RNA (6, 7). In ART-suppressed people with HIV (PWH), heterogeneous subsets of HIV-transcribing cells show varying degrees of progression through blocks to HIV transcriptional elongation, completion, and splicing (8, 9). Most of this RNA appears to be defective as a result of incomplete processivity and/or transcription from defective proviruses, but a small fraction of the HIV RNA appears to be intact (7).

These HIV-transcribing reservoirs likely have biologic and clinical significance. First, compared to HIV-uninfected people, ART-suppressed PWH show a higher mortality and increased incidence of diseases in many organ systems, which are thought to result from higher average levels of immune activation and inflammation (10). Although one study did not find an association between levels of Pol HIV RNA and immune activation (11), continued expression of some forms of HIV RNA, protein, or viral particles from defective and intact proviruses is thought to be a major cause of this immune activation (12). Second, one study found that levels of cell-associated Gag RNA in ART-suppressed PWH were higher in those who subsequently experienced virologic failure than in those who remained suppressed (13). Third, the subsets of infectious proviruses that spontaneously transcribe HIV RNA may have already overcome some barriers to HIV expression and may be poised to contribute to rebound after stopping ART. In support of this hypothesis, at least four studies have shown that levels of cell-associated HIV transcripts, including multiply-spliced Nef, unspliced Gag or Pol, and 5′ elongated HIV RNA, correlate with time to rebound of plasma viremia after ART interruption (14–17). Moreover, one small study found that in roughly half of individuals who stopped ART, p6-PR-RT sequences in the rebound virus matched those found in cell-associated HIV RNA from the blood just prior to ART interruption (18). Finally, cells that express HIV RNA and/or protein are likely more susceptible to many new therapies aimed at HIV cure, including those aimed at augmenting immune responses, killing cells expressing HIV proteins, or knocking out proviruses in accessible areas of chromatin.

Relatively few studies have investigated how the “active” HIV-transcribing reservoirs change with time on ART, as measured using longitudinal samples obtained from before and various times after ART. Most such studies have quantified a single form of cell-associated HIV RNA (CA HIV RNA)—typically either multiply spliced (12, 19) or unspliced (Gag or Pol) HIV RNA (6, 11, 20)—or both unspliced and multiply spliced HIV RNA (13, 21, 22), while two have sequenced various cell-associated HIV transcripts (6, 23). Moreover, very few studies have normalized the total levels of a given HIV transcript to the copies of the same proviral DNA region, which accounts for changes in HIV infection frequency on ART (due to clearance of infected cells) as well as possible changes in the relative frequency of proviral mutations that can affect HIV transcription and/or the ability to detect the corresponding HIV RNA region (24).

In a recent study, we measured the levels of different HIV transcripts and the corresponding HIV DNA regions/proviruses in longitudinal samples of CD4+ T cells obtained before ART and post-ART (6 months and 1 year) from 16 PWH who initiated ART during acute infection (24). Before ART, we found significant differences between the levels of different HIV RNA regions/transcripts that persisted after normalization to the corresponding HIV DNA regions, suggesting that even during untreated acute infection, some infected cells harbor blocks at various stages of HIV transcription (24). By 6 months after ART, we observed decreases in all HIV DNA regions/proviruses and HIV transcripts, but completed and mid-transcribed HIV RNA decayed faster than initiated or 5′ elongated HIV RNA, while intact HIV transcripts tended to decay faster than defective ones (24). These findings suggest that immune responses exert differential effects on infected cells transcribing different forms of HIV RNA. Between 6 months and 1 year on ART, we observed no further changes in HIV RNA or HIV RNA/DNA, suggesting possible attainment of an equilibrium (24).

It is unclear whether the same findings would hold true in the much larger population of PWH who initiate ART during chronic infection. Moreover, due to the lack of available samples after 1 year on ART in the acute cohort, we were unable to determine whether HIV DNA or RNA would continue to decline after much longer periods on ART. Most published studies that have prospectively measured CA-HIV RNA in longitudinal samples have been limited to 1 (21, 23, 25), 2 (12, 22), or 4–5 years of ART (19, 20). In these studies, multiply spliced or unspliced HIV RNA declined on ART, but no further decreases were observed after as few as 6–12 weeks (19, 22) to 1 year on ART (11, 13, 20). In one study that measured longitudinal levels of Gag HIV RNA in 3 PWH over 18–20 years, two individuals showed an initial decline in Gag RNA followed by a plateau, while the third showed a continual decline (6). In another study with follow-up for as much as 6–15 years on ART, Pol HIV RNA showed no further decrease after the first year on ART, while HIV DNA continued to decline after year 4 (11, 26).

**Fig 1:**
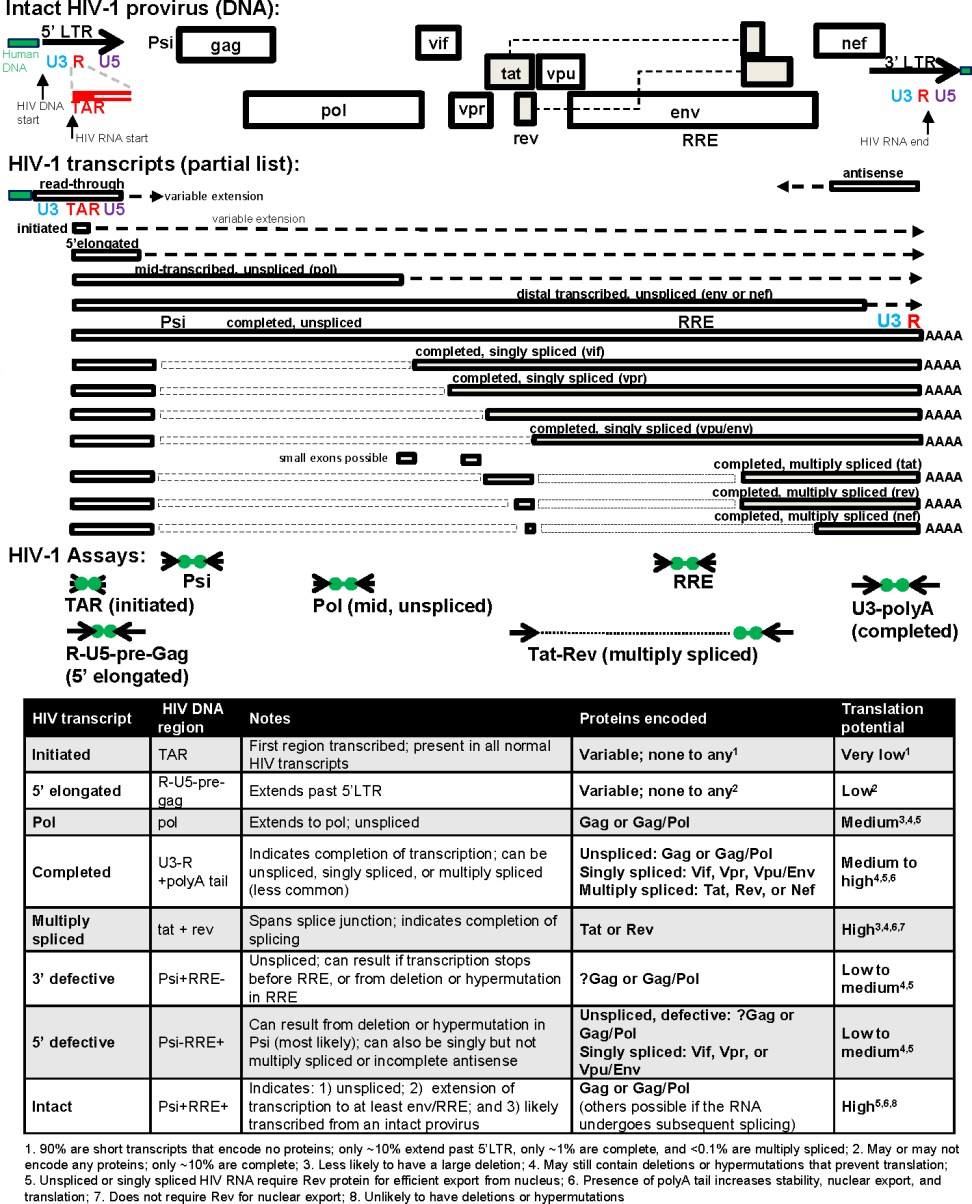
HIV-1 provirus, transcripts, assay locations, and translation potential. Shown are the genome organization of an intact HIV-1 provirus along with different types of HIV-1 transcripts (partial list), the location of assays used to quantify various HIV RNA (and sometimes HIV DNA) levels, and a table giving more information about the HIV transcripts quantified by these assays, including the possible proteins that may be encoded and a qualitative measure of translation potential (explained by numbered notes at bottom).

We hypothesized that different HIV transcripts would vary in their levels during untreated chronic infection, their rate of decline on ART, and whether they decrease to undetectable levels, reach a low but stable level, or continue to decline over the years on ART. In addition, we hypothesized that there would be differences between people who initiate ART during acute and chronic infection. To test these hypotheses, we measured levels of various HIV transcripts and their corresponding HIV DNA regions/proviruses (Fig. 1) in longitudinal samples obtained from 10 PWH during untreated chronic infection (T1) and up to four additional timepoints (T2–T5) covering up to 6–10 years on suppressive ART, and we compared these HIV levels to those measured previously in people who initiated ART during acute infection (24).

## MATERIALS AND METHODS

### Study participants

**Fig 2:**
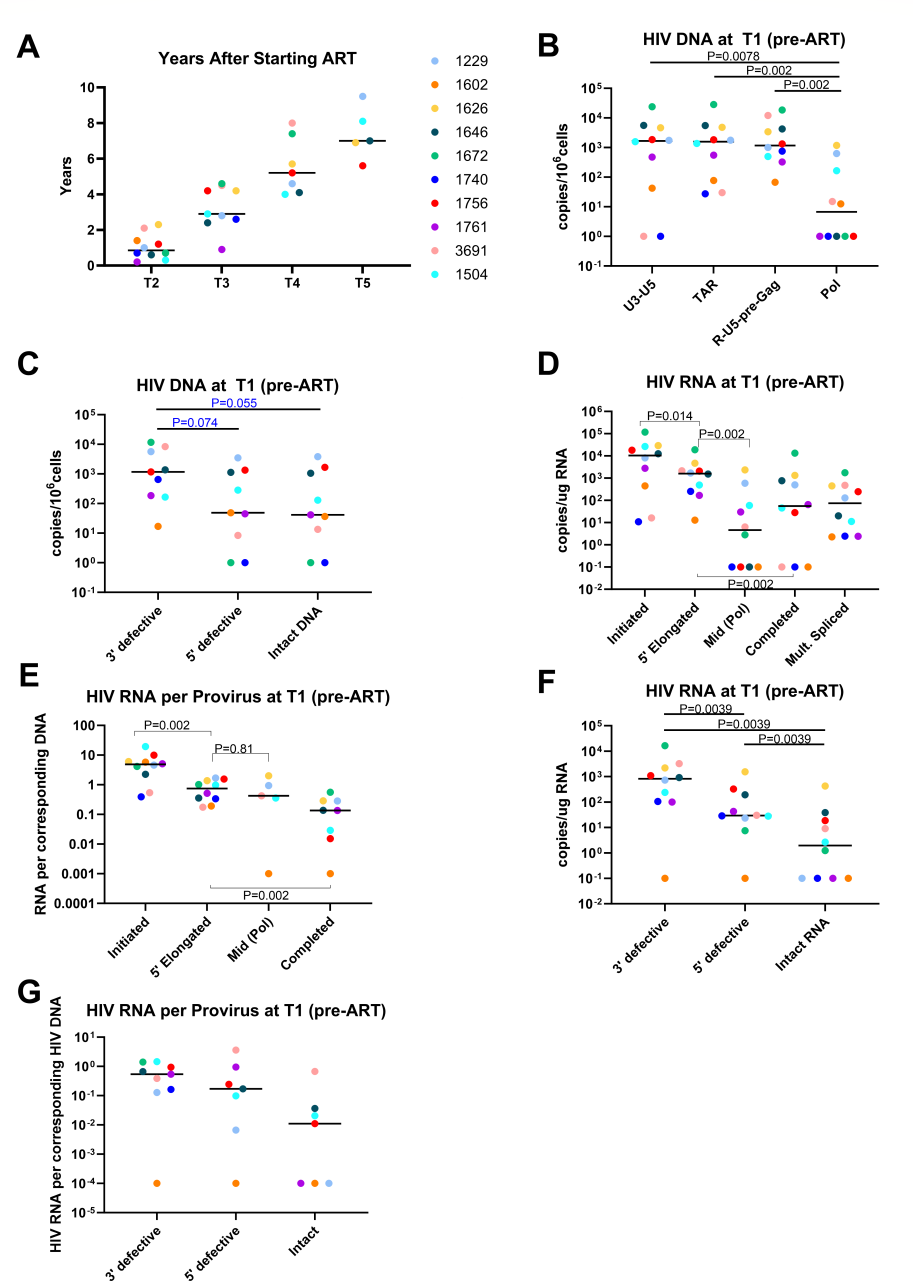
HIV-1 DNA and RNA levels during untreated chronic infection (T1). (**A**) The number of years after starting ART (set as time 0) is plotted for the sampling timepoints on suppressive ART (T2, T3, T4, and T5; x-axis). (**B**) Levels of U3-U5, TAR, R-U5-pre-Gag, and Pol HIV DNA were measured by ddPCR and normalized to copies per 10^6^ cells by DNA input into the PCR (assuming 1 µg of total DNA corresponds to 160,000 cells). (**C**) Levels of 3′ defective, 5′ defective, and intact HIV DNA were measured by ddPCR (IPDA). (**D**) Levels of initiated (TAR), 5′ elongated (R-U5-pre-Gag), mid-transcribed/unspliced (Pol), completed (PolyA), and multiply spliced (TatRev) HIV transcripts were measured by RT-ddPCR and normalized to cellular transcription (1 µg of total RNA, which corresponds to 10^6^ cells). (**E**) Levels of initiated, 5′ elongated, mid (Pol), and completed HIV RNA were normalized to levels of the same or corresponding HIV DNA region. (**F**) Levels of 3′ defective, 5′ defective, and intact HIV RNA were measured by dd-RT-PCR (Intact Viral RNA Assay) and normalized to cellular transcription (1 µg of total RNA, which corresponds to 10^6^ cells). (**G**) Levels of 3′ defective, 5′ defective, and intact HIV RNA were normalized to levels of the same or corresponding HIV DNA region. Horizontal lines indicate medians; different colors indicate individual study participants. *P*-values (two-tailed) were calculated using the Wilcoxon matched-pairs signed rank test.

The study participants included 10 individuals from the UCSF SCOPE HIV cohort who were diagnosed with HIV during chronic infection and followed up longitudinally between June 2008 and July 2022. For the current study, blood samples were taken prior to ART initiation (time point 1, T1) and up to four timepoints (T2, T3, T4, and T5) on suppressive ART. The variation in sampling intervals is displayed in Fig. 2A (median years after ART start: T2 = 0.9; T3 = 2.9; T4 = 5.2; and T5 = 7). Samples were available from 10 participants at T1, T2, and T3, with T4 samples available from 8 of these participants and T5 samples available from 5 of these participants. Peripheral blood sampling at each visit was performed to measure plasma HIV RNA (Abbott Real-Time PCR assay, limit of detection <40 copies/mL) and isolate peripheral blood mononuclear cells (PBMC), which were cryopreserved. All study participants provided written informed consent.

### Nucleic acid isolation

Cryovials containing 10 × 10^6^ PBMCs were thawed in a 37°C water bath, diluted in PBS with 2% FBS, and centrifuged at 250 *× g* for 5 min to pellet cells. CD4+ T cells were isolated from PBMCs using the EasySep Human CD4+ T Cell Enrichment Kit (StemCell Technologies). Total cellular DNA and RNA were extracted in parallel from CD4+ T cells using the Qiagen AllPrep DNA/RNA/miRNA Universal Kit with on-column DNase I treatment of the RNA and the manufacturer’s modification to enhance recovery of short transcripts. RNA and DNA concentrations were measured using UV spectrophotometry (NanoDrop One, Thermo Fisher).

### Quantification of HIV DNA by ddPCR

Levels of different HIV DNA regions, including the U3-U5 Long Terminal Repeat (LTR), *Trans* Activation Response (TAR) region, R-U5-pre-Gag region, and Pol region, were quantified using droplet digital PCR (ddPCR) as described previously (8). HIV DNA copies were normalized to cell numbers according to the mass of DNA input per well (calculated from the DNA concentration and input volume) (27). The Intact Proviral DNA Assay (IPDA; duplex ddPCR for the HIV Packaging Signal [Psi] and Rev Response Element [RRE] regions) was performed as described previously (24, 28). Each sample was tested in at least two replicate ddPCR wells with a maximum DNA input of 750 ng per well.

### Reverse transcription and quantification of HIV RNA

Total initiated HIV (TAR) transcripts were quantified by a three-step polyadenylation-RT-ddPCR as described previously (8, 29). HIV 5′ elongated (R-U5-pre-Gag), mid-transcribed (Pol), completed (U3-PolyA), and multiply spliced (Tat-Rev) transcripts were quantified by a two-step RT-ddPCR as described previously (8, 24). The Intact Viral RNA Assay (IVRA; duplex assay for the HIV Packaging Signal [Psi] and Rev Response Element [RRE] RNA) was performed using a one-step droplet digital reverse transcriptase PCR (dd-RT-PCR) as described previously (7, 24). Each sample was tested in at least two replicate ddPCR wells with a maximum RNA input of 300 ng per well. HIV RNA copies were normalized to total cellular transcription (1 µg of RNA, corresponding to about 10^6^ cells) according to the RNA concentration, the volumes used in the RT, and the fraction going into each ddPCR well (8).

### Statistical analysis

The Wilcoxon signed rank test was used to compare levels of different HIV DNA regions/proviruses, HIV transcripts, and ratios (HIV RNA/RNA or HIV RNA/DNA) within time points and between time points. The Mann-Whitney test was used to compare levels of HIV targets in this study to the same HIV targets measured at the same or corresponding timepoints from a prior study of people who initiated ART during acute infection (24). For calculating the median and *P* values, samples with no detectable HIV were assigned values that were lower than the lowest measured value, including 0.1 copy for HIV RNA, 1.0 copy for HIV DNA, and ≤0.001 for certain HIV ratios for which the numerator was not detected. Correlations were assessed using the Spearman r and *P* values. All statistics were performed using GraphPad Prism (Version 9.5.1). *P*-values that were corrected for multiple comparisons using the Benjamini–Hochberg method are outlined in Table S1.

## RESULTS

### Study participants and study design

CD4+ T cells were isolated from longitudinal blood samples from 10 PWH during untreated chronic infection (T1) and from 4 timepoints on suppressive ART (median years after ART start: T2 = 0.9; T3 = 2.9; T4 = 5.2; and T5 = 7; Fig. 2A). The median time from HIV diagnosis to the start of ART was 3.4 years (range: 0.5–25; Table S1). The median viral load at T1 was 7,081 copies/mL (range: 1,483–235,492). The median CD4+ T-cell count at T1 was 535 cells/µL (range: 336–972), and the median CD4% at T1 was 25.5% (range: 15–37%). After starting ART, all individuals had a viral load of <40 copies/mL at T2, and all viral loads remained <40 copies/mL from T2 to T5.

### Differences in HIV DNA levels before ART

Levels of various HIV DNA regions (corresponding to different HIV RNA transcripts) were measured in blood CD4+ T cells before (T1) and after initiation of ART (T2–T5), including (i) the U3-U5 LTR (this HIV DNA region contains the sequence targeted in the assay for U3-polyadenylated or “completed” HIV transcripts, and both assays share the same forward primer and probe); (ii) the TAR region (corresponding to initiated HIV RNA); (iii) the R-U5-pre-Gag region (corresponding to 5′ elongated HIV RNA); and (iv) the Pol region (corresponding to mid-transcribed, unspliced HIV RNA). Before ART (T1), HIV DNA levels varied greatly between study participants (>3 log_10_; Fig. 2B and C). The levels of U3-U5, TAR, and R-U5-pre-Gag HIV DNA regions (which all include part of the LTR) were all comparable to each other, but all three were present at higher levels than the Pol HIV DNA region (*P* = 0.0078, *P* = 0.002, *P* = 0.002, respectively; Fig. 2B), likely due to a greater frequency of mutations in Pol. Compared to people with untreated acute infection, participants in chronic infection showed lower levels of TAR DNA (*P* = 0.021) and a trend toward lower levels of Pol (*P* = 0.071) and U3-U5 DNA (*P* = 0.077; Fig. S1A), suggesting some clearance of infected cells over time during untreated infection.

**Fig 3:**
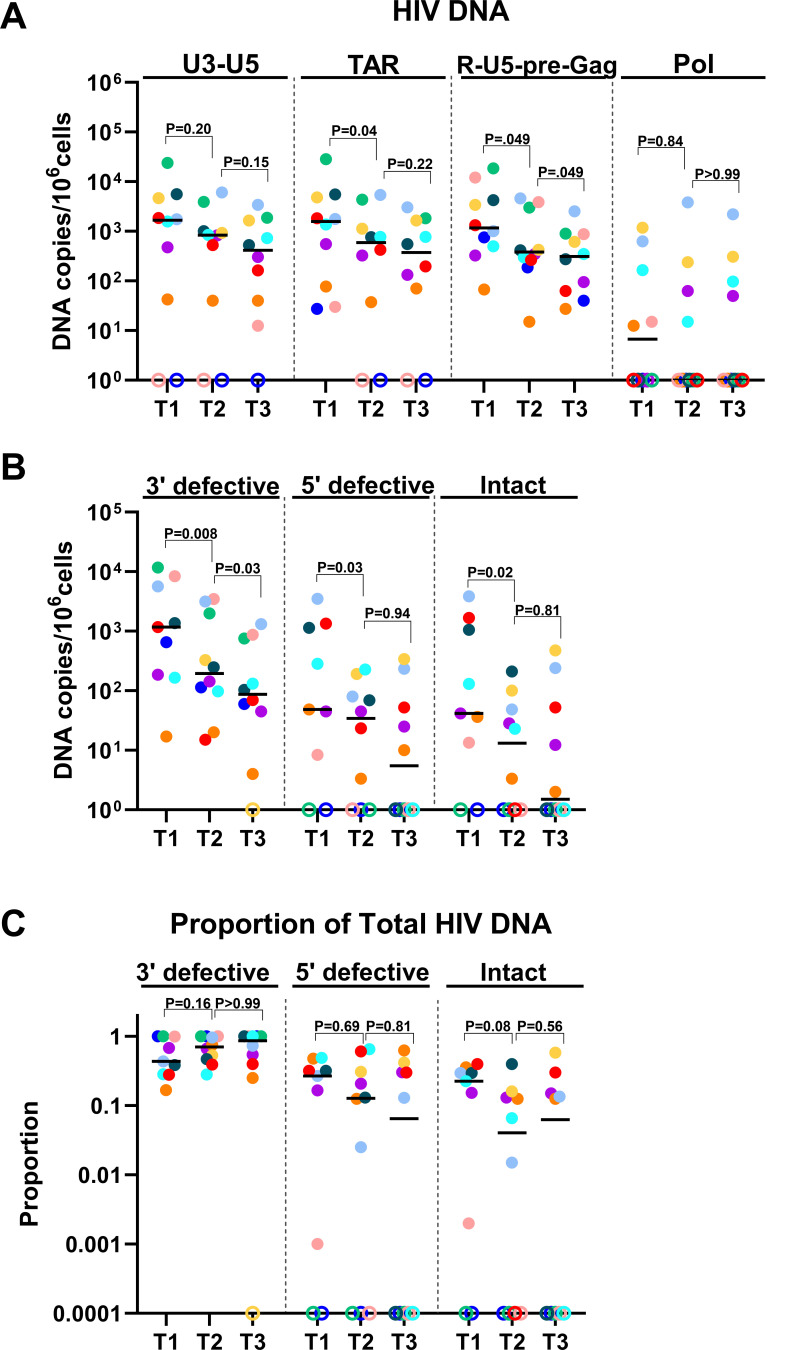
Differential changes in different HIV-1 DNA regions or proviruses after initiation of ART. (**A**) Levels of U3-U5, TAR, R-U5-pre-Gag, and Pol HIV DNA regions were measured by ddPCR in longitudinal samples from before ART (T1) and a median of 1 year (T2) and 3 years (T3) after ART. (**B**) Levels of 3′ defective (Psi+ RRE−), 5′ defective (Psi− RRE+), and intact (Psi+ RRE+) HIV proviruses were measured by ddPCR (IPDA). (**C**) Proportion of the total HIV DNA as measured by ddPCR (IPDA). Horizontal lines indicate medians, different colors indicate individual study participants, and open circles indicate undetectable values. *P*-values (two-tailed) were calculated using the Wilcoxon signed rank test.

During untreated chronic infection, levels of 3′ defective HIV DNA tended to be greater than either 5′ defective (*P* = 0.074) or intact proviruses (*P* = 0.055; Fig. 2C). Intact HIV DNA constituted a median of 22.5% of the total HIV DNA at T1 (Fig. 2C and 3C). Compared to people with untreated acute infection, participants in chronic infection showed lower levels of 5′ defective HIV DNA (*P* = 0.011; Fig. S1B).

### Before ART, the blocks to HIV transcription differed between chronic and acute infection

Levels of initiated (TAR), 5′ elongated (R-U5-pre-Gag), mid-transcribed/unspliced (Pol), completed (PolyA), and multiply spliced (Tat-Rev) HIV transcripts were measured by RT-ddPCR at times T1–T5 and normalized to total cellular transcription (1 µg of RNA, corresponding to ~10^6^ CD4+ T cells). During untreated chronic infection (T1), we observed an excess of initiated over 5′ elongated HIV RNA (median elongated/initiated = 0.16; *P* = 0.014) and of 5′ elongated over completed HIV RNA (median completed/elongated = 0.03; *P* = 0.002; Fig. 2D), suggesting blocks to HIV transcriptional elongation and completion. However, in contrast to untreated PWH during acute infection (24), we observed no difference between completed and multiply spliced HIV RNA (Fig. 2D). Compared to people with untreated acute infection, participants in chronic infection showed lower total levels of Pol (unspliced, mid-transcribed) HIV RNA (*P* = 0.0026) and a trend toward lower levels of initiated (*P* = 0.067) and completed HIV RNA (*P* = 0.077; Fig. S1C).

To account for differences in infection frequency as well as the relative frequencies of proviral mutations at different DNA regions, we normalized levels of each HIV RNA to levels of the same or corresponding HIV DNA region. In untreated chronic infection, the differences between levels of initiated, elongated, and completed HIV transcripts remained after normalization of each HIV RNA to the corresponding HIV DNA region (Fig. 2E), further suggesting that differences in these HIV transcript levels are due to transcriptional blocks rather than differences in the frequency of mutations at the corresponding DNA regions. Compared to people in untreated acute infection, participants in chronic infection showed a trend toward higher levels of initiated HIV RNA/DNA (HIV transcription initiation) but no significant difference in levels of elongated, Pol, or completed HIV RNA per provirus (Fig. S1E).

To evaluate the progression through blocks to HIV transcriptional elongation, completion, and splicing in a manner that is independent of infection frequency or progression through previous blocks to HIV transcription, we also calculated the ratios of one HIV RNA to another to measure the extent of 5′ elongation (elongated/initiated), completion (completed/elongated), and multiple splicing (multiply spliced/completed). During untreated chronic infection, a median of approximately 10% of HIV transcripts progressed through each stage of 5′ elongation, completion, and splicing (Fig. S1G). HIV transcriptional completion tended to be lower than observed during untreated acute infection (*P* = 0.072; Fig. S1G).

We also used the Intact Viral RNA Assay (IVRA) to measure levels of 3′ defective, 5′ defective, and intact HIV RNA during untreated chronic infection (T1). Levels of 3′ defective HIV RNA were significantly greater than 5′ defective or intact HIV RNA (*P* = 0.0039 for both), and 5′ defective HIV RNA was more abundant than intact HIV RNA (*P* = 0.0039; Fig. 2F). The median ratio of intact to total HIV RNA was 0.27% (Fig. 2F and 5D), which is 100-fold lower than the ratio of intact to total HIV DNA at T1 (22.5%). 5′ defective HIV RNA levels were lower than observed previously in untreated acute infection (*P* = 0.045), and the median level of intact HIV RNA tended to be lower (*P* = 0.11; Fig. S1D). The median ratio of intact HIV RNA to intact HIV DNA was ~0.01 (Fig. 2G), suggesting that at most 1% of intact proviruses are making intact HIV RNA during chronic infection. This ratio of intact HIV RNA/intact HIV DNA tended to be lower (*P* = 0.066) than that observed during untreated acute infection (median of ~0.1; Fig. S1F), suggesting that over time from acute to chronic infection, there is a selection against intact proviruses transcribing intact HIV RNA.

### ART causes differential decreases in HIV DNA regions/proviruses

After initiation of ART, we observed statistically significant reductions in the levels of TAR and R-U5-pre-Gag HIV DNA (median T1/T2 = 4.3 for both, *P* = 0.037 and *P* = 0.049, respectively) but not U3-U5 or Pol DNA (Fig. 3A). From T2 to T3, only the R-U5-pre-Gag HIV DNA continued to decrease (median T2/T3 = 2.6, *P* = 0.049; Fig. 3A). Extended time on ART further reduced the levels of R-U5-pre-Gag HIV DNA (median T3/T4 = 2.2, *P* = 0.039; Fig. S2A).

To account for variations in sampling intervals between individuals (Fig. 2A), we also calculated the change in each HIV DNA region divided by the change in time between timepoints (for T1 vs. T2, we used the time since the start of ART) and compared the resulting slopes. From ART start to T2, the slope decline in TAR HIV DNA was greater than U3-U5 DNA (*P* = 0.049), and the slope changes in both TAR and R-U5-pre-Gag HIV DNA were greater than that of Pol HIV DNA (*P* = 0.014 for both; Fig. S2C). From T2 to T3, the slope decrease in R-U5-pre-Gag HIV DNA continued to be greater than that of Pol HIV DNA (*P* = 0.014; Fig. S2D).

Intact (Psi+ RRE+) and defective (Psi− RRE+ and Psi+ RRE−) HIV DNA were measured by ddPCR (IPDA). From T1 to T2, 3′ defective (Psi+ RRE−), 5′ defective (Psi− RRE+), and intact (Psi+ RRE + ) HIV DNA decreased significantly (median T1/T2 = 2.1, 4.8, and 5.4, respectively; *P* = 0.0078, 0.031, 0.016, respectively; Fig. 3B). From T2 to T3, a significant decrease was observed only for 3′ defective HIV DNA (median T1/T2 = 2.5; *P* = 0.027; Fig. 3B). No further reductions were observed from T3 to T4 or T4 to T5 (Fig. S3A and B). From T1 to T2, ART tended to reduce the ratio of intact to total HIV DNA from a median of 22.5% to approximately 4% (*P* = 0.078; Fig. 3C).

Next, we calculated the slope change in HIV DNA over the change in time. From ART start to T2, we did not detect significant differences between the slope decreases in defective or intact HIV DNA, although the median slope was greater for 3′ defective HIV DNA than either 5′ defective or intact HIV (Fig. S3C). However, from T2 to T3, the slope decrease in 3′ defective HIV DNA was greater than 5′ defective HIV DNA (*P* = 0.027) and tended to be greater than intact HIV DNA (*P* = 0.065; Fig. S3D).

### ART causes differential decay of various HIV transcripts

**Fig 4:**
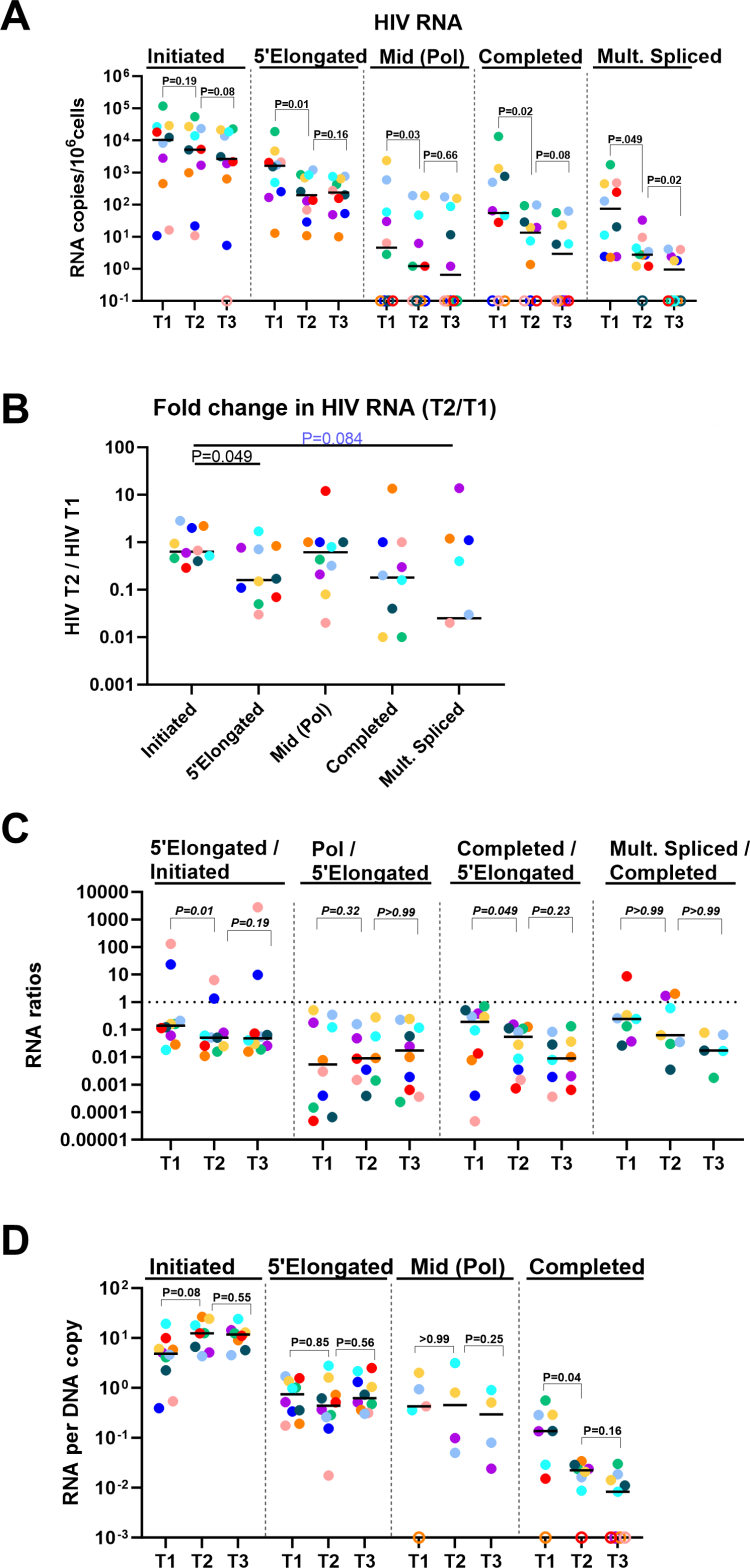
Differential changes in different HIV-1 transcripts after initiation of ART. (**A**) Levels of initiated (TAR), 5′ elongated (R-U5-pre-Gag), mid-transcribed/unspliced (Pol), completed (PolyA), and multiply spliced (Tat-Rev) HIV transcripts were measured by RT-ddPCR in longitudinal samples from before ART (T1) and a median of 1 year (T2) and 3 years (T3) after ART. HIV RNA levels were normalized to cellular transcription (1 µg of total RNA, which corresponds to 10^6^ cells). (**B**) The fold change in each HIV transcript was expressed by the ratio of the levels at each HIV transcript at T2 divided by the level of the same HIV transcript at T1. Of note, a lower T2/T1 ratio corresponds to a larger fold decrease. (**C**) The ratios of one HIV RNA to another were used to evaluate the progression through HIV transcriptional elongation, mid-transcription, completion, and splicing before (T1) and after initiation of ART (T2–T3). Ratios are independent of effects at prior stages of HIV transcription and independent of infection frequency or normalization to cell numbers. Shown are the proportions of (i) all HIV transcripts that were elongated (5′ elongated/initiated); (ii) elongated HIV transcripts that were mid-transcribed (Pol/elongated) or (iii) completed (completed/elongated); and (iv) completed transcripts that were multiply spliced (multiply spliced/completed). (**D**) To account for possible changes over time in the infection frequency or the relative frequencies of proviral mutations at different HIV DNA regions, the levels of each HIV transcript were normalized to levels of the corresponding HIV DNA region at the same time point. Horizontal lines indicate medians, different colors indicate individual study participants, and open circles indicate undetectable values. *P*-values (two-tailed) were calculated using the Wilcoxon signed rank test.

From T1 (pre-ART) to T2, we observed reductions in 5′ elongated (median T1/T2 = 6; *P* = 0.014), mid-transcribed Pol (T1/T2 = 2; *P* = 0.031), completed (T1/T2 = 6; *P* = 0.016), and multiply spliced HIV RNA (T1/T2 = 43, *P* = 0.049) but not initiated HIV RNA (Fig. 4A). From T2 to T3, multiply spliced HIV RNA declined further (*P* = 0.020), and initiated and completed HIV RNA tended to decrease (*P* = 0.084 and *P* = 0.078; Fig. 4A). No further reductions in any type of RNA were observed from T3 to T5 (Fig. S4A and B). As we observed before ART, differences in the levels of various HIV RNA regions persisted at T2, with an excess of initiated over 5′ elongated (median elongated/initiated = 0.04; *P* = 0.0098) and of 5′ elongated over completed HIV RNA (median completed/elongated = 0.07; *P* = 0.002) but no difference between completed and multiply spliced HIV RNA (Fig. S5A).

To determine whether there were significant differences between the decay rates of various HIV transcripts, we calculated the fold change in each transcript by dividing its value at T2 by that at T1 and then compared this ratio with the fold changes of the other transcripts. From T1 to T2, the fold decrease in 5′ elongated HIV RNA was greater (lower T2/T1) than that of initiated HIV RNA (*P* = 0.049), and the fold change in multiply spliced HIV RNA tended to be greater (lower T2/T1) than that of initiated HIV RNA (*P* = 0.084; Fig. 4B).

To account for variations in sampling intervals between individuals (Fig. 2A), we also calculated the slope change in each HIV transcript, as measured by the change in each HIV RNA divided by the change in time between timepoints. From ART start to T2, the slope decline in 5′ elongated HIV transcripts was greater than that of mid-transcribed (Pol) HIV transcripts (*P* = 0.037; Fig. S4C). No significant differences were observed from T2 to T3 (Fig. S4D).

To evaluate the progression through blocks to HIV transcriptional elongation, completion, and splicing, we also calculated the ratio of one HIV RNA to another at a given time point and compared it between time points. The ratio of elongated/initiated HIV RNA decreased from T1 (median = 0.14) to T2 (median = 0.051; *P* = 0.014; Fig. 4C), suggesting a decrease in HIV transcriptional elongation. Likewise, the ratio of completed/elongated HIV RNA decreased from T1 to T2 (0.055 vs. 0.19; *P* = 0.049; Fig. 4C), suggesting a decrease in completion. No further reductions were observed at T3 (Fig. 4C) or T4-5 (Fig. S5B and C).

To account for differences in how the relative frequencies of various proviral regions or mutations at these regions may change over time, we also normalized each HIV transcript to levels of the corresponding HIV DNA region at the same time point and then compared the HIV RNA/DNA across time points. From T1 to T2, ART reduced completed (PolyA) HIV RNA per provirus (median T1/T2 = 10; *P* = 0.039), suggesting that the decrease in completed HIV RNA is not due to a decrease in the corresponding HIV DNA (Fig. 4D). By contrast, we did not detect a decrease in elongated or mid-transcribed HIV RNA per provirus, suggesting that the reductions in these HIV transcripts may have been driven by changes in the corresponding HIV DNA regions. From T1 to T2, we observed a trend toward an increase in initiated (TAR) RNA/DNA (*P* = 0.078, Fig. 4D), as was previously observed in a cohort of acutely infected PWH (24). No further reductions in any type of RNA per provirus were observed at T3 (Fig. 4D) or T4-5 (Fig. S6A and B). When comparing the slope changes in the ratio of each HIV RNA to the corresponding HIV DNA over time, we did not detect significant differences from ART start to T2, or between T2 and T3 (Fig. S6C and D).

**Fig 5:**
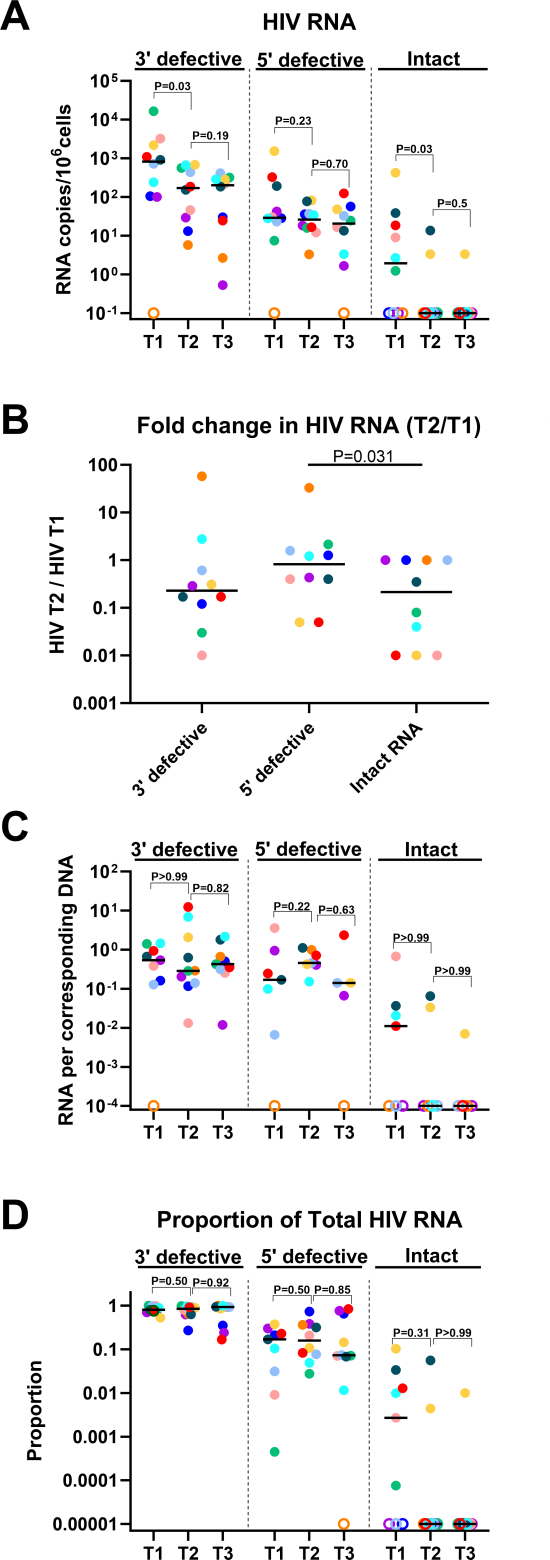
Differential changes in defective and intact HIV-1 transcripts after initiation of ART. (**A**) Levels of 3′ defective (Psi +RRE−), 5′ defective (Psi−RRE+), and intact (Psi +RRE + ) HIV transcripts were measured by dd-RT-PCR in longitudinal samples from before ART (T1) and a median of 1 year (T2) and 3 years (T3) after ART. HIV RNA levels were normalized to cellular transcription (1 µg of total RNA, which corresponds to 10^6^ cells). (**B**) The fold change in each HIV transcript was expressed by the ratio of the levels at each HIV transcript at T2 divided by the level of the same HIV transcript at T1. Of note, a lower T2/T1 ratio corresponds to a larger fold decrease. (**C**) To account for possible changes over time in the infection frequency or the relative frequencies of proviral mutations at different HIV DNA regions, the levels of each HIV transcript were normalized to levels of the corresponding HIV provirus at the same time point. (**D**) Proportion of the total HIV RNA as measured by dd-RT-PCR. Horizontal lines indicate medians, different colors indicate individual study participants, and open circles indicate undetectable values. *P*-values (two-tailed) were calculated using the Wilcoxon signed rank test.

Using the Intact Viral RNA Assay (7), we also examined the effect of ART on intact and defective HIV RNA. From T1 to T2, ART reduced 3′ defective and intact HIV RNA (median T1/T2 = 4.7 and 7.6; *P* = 0.0027 and *P* = 0.031; Fig. 5A). The fold decrease in intact HIV RNA was greater than that of 5′ defective HIV RNA (median T2/T1 = 0.22 vs. 0.83; *P* = 0.031; Fig. 5B). When calculating the change in HIV RNA over time from ART start to T2, the slope change in 3′ defective HIV RNA was greater than intact HIV RNA (*P* = 0.049) and tended to be greater than 5′ defective HIV RNA (*P* = 0.065; Fig. S7A).

Unlike defective HIV RNA, the levels of intact HIV RNA were reduced to undetectable levels in 8 out of 10 individuals by T2 (Fig. 5A) and were undetectable in all individuals at T4–T5 (Fig. S7C and D). No further declines in intact or defective HIV RNA were observed from T2 to T3 (Fig. 5A) or after T3 (Fig. S7C and D). When intact and defective HIV RNA were normalized to levels of the corresponding HIV DNA, no significant changes were observed from T1 to T2 or T2 to T3 (Fig. 5C). However, ART reduced the median ratio of intact/total HIV RNA below the limit of detection (Fig. 5D), further suggesting preferential selection against cells transcribing intact HIV RNA.

### Differences between acute-treated and chronic-treated PWH after 1 year of ART

We also compared HIV DNA and RNA levels at T2 (~1 year after ART) to those previously measured at 1 year post-ART (the last available timepoint) in people who initiated ART during acute infection. After 1 year of ART, levels of R-U5-pre-Gag HIV DNA were higher in those who initiated ART during chronic infection (*P* = 0.037; Fig. S8A), while we did not detect differences in other HIV DNA regions (Fig. S8A) or in defective or intact HIV DNA (Fig. S8B). No significant differences were detected in total levels of initiated, 5′ elongated, Pol, completed, or multiply-spliced HIV RNA (Fig. S8C), or in levels of these transcripts per corresponding provirus (Fig. S8E) or the progression through stages of HIV transcription (Fig. S8G). However, individuals who started ART during chronic infection had higher levels of 5′ defective HIV RNA (*P* = 0.03) and a trend toward higher levels of 3′ defective HIV RNA (*P* = 0.084; Fig. S8D), while intact HIV RNA was infrequently detected in either group. After normalization to the corresponding HIV DNA, the chronic cohort still had higher levels of 5′ defective (*P* = 0.002) but not 3′ defective HIV RNA per provirus (Fig. S8F). These findings suggest that the delay of ART until chronic infection may reduce immune responses that allow persistence of certain proviruses, including those that have an intact 5′ LTR and/or those transcribing defective HIV RNA.

### The plasma viral load at T1 correlates with some HIV RNAs and DNAs at T1 but not at T2 or T3

During untreated chronic infection (T1), we observed significant correlations between the plasma viral load and both defective HIV RNA and R-U5-pre-Gag DNA (*P* = 0.035 and *P* = 0.023, respectively; Fig. S9A; Table S2). The viral load also tended to correlate with 5′ elongated and multiply spliced HIV RNA (both *P* ≤ 0.06). By contrast, none of the HIV RNAs or DNAs at T2 or T3 correlated significantly with the viral load at T1 (Fig. S9B and C). At T1, completed HIV RNA correlated with initiated HIV RNA (*P* = 0.0079), and both correlated with levels of the U3-U5 and TAR HIV DNA regions (all *P* ≤ 0.002). Multiply spliced HIV RNA correlated with 5′ elongated HIV RNA (*P* = 0.00001), defective RNA (*P* = 0.00005), and R-U5-Pre-Gag DNA (*P* = 0.0005), while intact HIV RNA correlated only with defective HIV RNA (*P* = 0.034; Fig. S9A).

At T2, initiated, 5′ elongated, and completed HIV RNA correlated with each other, and also with U3-U5 DNA and TAR DNA (all *P* < 0.05), while none of these correlated with multiply spliced HIV RNA, intact HIV RNA, or intact HIV DNA (Fig. S9B). Intact HIV RNA correlated only with multiply spliced HIV RNA (negative correlation; *P* = 0.044) and intact DNA (positive correlation; *P* = 0.011).

At T3, initiated, 5′ elongated, completed, and defective HIV RNA correlated with each other, and also with U3-U5 DNA and TAR DNA (all *P* < 0.05), while none of these correlated with multiply spliced HIV RNA, intact HIV RNA, or intact HIV DNA (Fig. S9C). Of the different types of HIV DNA, U3-U5 and TAR HIV DNA correlated significantly with all HIV transcripts except for multiply spliced and intact HIV RNA during both suppressed time points (T2-T3; all *P* < 0.05, except *P* = 0.08 for the correlation between Pol and U3-U5 HIV DNA at T2; Fig. S9B and C).

## DISCUSSION

To our knowledge, this study is the first to measure progression through various blocks to HIV transcription and levels of intact and defective HIV RNA (along with the corresponding HIV DNA regions/proviruses) in untreated chronic infection or longitudinal samples from more than 1 year (and up to 10 years) after the start of ART. During untreated chronic infection and all timepoints on ART, levels of Pol HIV DNA were much lower than levels of the 5′ LTR region (R-U5-pre-Gag) and less than half the level of assays targeting both LTR regions (TAR, U3-U5). Since the efficiency of the Pol assay is not less than that of the other assays (8), the lower levels of Pol HIV DNA are likely due to a relatively higher frequency of proviral mutations in Pol (1, 2, 28) compared to the LTR regions. These results accord with those observed before and after ART initiation during acute infection (24). However, the median levels of most HIV DNA regions/proviruses were lower in untreated chronic infection than acute infection, likely due to immune-mediated clearance and/or the accumulation of additional proviral mutations over time from acute to chronic infection.

During untreated chronic infection, we also found significant differences between levels of initiated, 5′ elongated, and completed HIV transcripts, which are not explained by differences in assay efficiencies (8) or the rates at which these HIV transcripts degrade in CD4+ T cells (9). The difference between levels of these HIV transcripts persisted after normalization to the corresponding HIV DNA regions, suggesting that they are not explained by differences in the relative frequency of proviral mutations at the corresponding HIV DNA regions. Instead, the differences between the levels of initiated, 5′ elongated, and completed HIV RNA/DNA suggest that even during untreated chronic HIV infection, there are blocks to HIV transcription (elongation, completion) in some infected cells and/or immune selection against cells making elongated and completed transcripts. The difference between initiated and elongated HIV RNA/DNA accords with results from people in untreated acute infection (24). However, in contrast to our findings in acute infection (24), we observed no difference between levels of completed and multiply spliced HIV RNA during untreated chronic infection. The lower number of samples from chronic infection (10, versus 16 in the acute treatment study) may have resulted in lower power to detect differences, but it is also possible that there is less block to HIV multiple splicing or a greater progression through HIV splicing during chronic infection.

We also found other differences between untreated chronic and acute infections that have not previously been reported. Total levels of initiated HIV transcripts tended to be lower in chronic infection, but when we corrected for levels of HIV DNA, we actually observed the opposite trend toward higher levels of initiated HIV transcripts per provirus (HIV transcriptional initiation). Given the vast excess of initiated over 5′ elongated HIV RNA, most of these initiated transcripts are likely to be short HIV transcripts limited to the TAR loop or 5′ LTR, which may accumulate over time during untreated infection. Individuals in chronic infection also had lower total levels of Pol HIV RNA and 5′ defective HIV RNA, as well as a trend toward lower levels of complete HIV RNA. These three differences were no longer significant after normalization to HIV DNA, suggesting that differences in HIV DNA (infection frequency or proviral mutations) were contributing. By contrast, the ratio of completed to elongated HIV RNA (which is independent of HIV DNA) tended to be lower in chronic infection, suggesting less completion of HIV transcription and/or preferential clearance of cells making completed HIV transcripts over time.

In accord with these results, the median ratio of intact HIV RNA to intact HIV DNA during untreated chronic infection was only 0.01. This low ratio does not seem to be the result of assay sensitivity or cell input, since we have previously shown that the Intact Viral RNA Assay (IVRA) can detect as few as 1–10 copies of intact HIV RNA (7), and the cell input into the IVRA (≥600 ng RNA≈600,000 cells) was no less than the IPDA (≥1,500 ng DNA≈240,000 cells). Therefore, the intact HIV RNA/intact HIV DNA ratio of 0.01 implies that, despite ongoing viremia, no more than 1% of intact proviruses are transcribing intact HIV RNA, and the remaining 99% have blocks at some stage of HIV transcription. Some of these cells with intact proviruses but not intact HIV RNA could still be making HIV transcripts that terminate prematurely prior to the RRE (as a result of blocks to HIV transcriptional elongation or completion), while others could be transcribing no HIV RNA (as a result of blocks to initiation). We are unable to distinguish among these possibilities, and each could be operating in different cells. However, given the large proportion of total HIV transcripts that appear to be blocked at the stages of HIV transcriptional elongation and completion, we suspect that these blocks are also operating in many cells with intact proviruses. The ratio of intact HIV RNA/intact HIV DNA also tended to be lower in chronic infection than in acute infection, with a 10-fold difference in the median levels (0.01 vs. 0.1). This difference could indicate that there is a progressive decrease in HIV transcription from intact proviruses, and/or progressive selection against intact proviruses making intact HIV RNA, over time from untreated acute to chronic infection.

After ART initiation during chronic infection, we observed a decrease in the TAR and R-U5-pre-Gag HIV DNA regions, but no decrease in U3-U5 or Pol HIV DNA, from T1 to T2 (~1 year post-ART). These findings contrast with those in acute-treated PWH, in whom we observed a significant decrease in all 4 of these HIV DNA regions over a shorter time period (from before ART to 6 months post-ART) (24). While the power to detect changes was lower in the chronic-treated cohort, it may also be that the delay of ART until chronic infection results in less clearance of HIV-infected cells (19), and/or that differences in these HIV DNA regions contribute to differences in the clearance of infected cells. After initiation of ART and subsequent suppression of plasma viremia at T2, the levels of R-U5-pre-Gag HIV DNA continued to decline from T2 to T3 (~3 years) and T3 to T4 (~5 years), while we observed no significant decrease in TAR, U3-U5, or Pol HIV DNA over the same time periods. This difference, which has not previously been reported, suggests that there is a more sustained immune selection against proviruses with the R-U5-pre-Gag region, which may have an intact (non-mutated) 5′ end and may be more likely to support HIV transcriptional elongation.

Using the IPDA to quantify intact and defective HIV DNA, we also found evidence for differential rates of clearance and achievement of long-term stability. From T1 to T2, we found significant decreases in 5′ defective, 3′ defective, and intact HIV DNA, as observed previously in acute-treated PWH (24). From T2 to T3, 3′ defective (Psi +RRE−) HIV DNA continued to decline, while we did not detect a significant decrease in intact or 5′ defective HIV DNA. While the lower starting levels of intact and (to a lesser extent) 5′ defective proviruses may have made it more difficult to detect further decreases, it is also possible that there was differential selection against the various types of proviruses. From T3 to T4 (~5 years) and T4 to T5 (~7 years), we did not detect any further decrease in either type of defective provirus or intact proviruses, in accord with some studies (30, 31) but not others (26, 32). This finding suggests the attainment of an equilibrium at which time any further clearance of infected cells is matched by proliferation. However, it is also worth noting that we had less of an ability to detect further changes because of the progressively lower levels of all proviruses and the smaller number of participants with samples from all five timepoints.

From T1 to T2, ART initiation led to significant decreases in 5′ elongated, Pol, completed, and multiply spliced HIV RNA, as we had observed previously in people who initiated ART during acute infection (24). However, in contrast to the acute-treated PWH, who showed a decrease in total initiated HIV transcripts after 6 months of suppressive ART (24), we observed no such decrease in the chronic-treated PWH despite a longer duration between T1 and T2. The fold decrease in elongated HIV RNA was greater than that of initiated HIV RNA, and the fold change in multiply spliced HIV RNA tended to be greater than that of initiated HIV RNA, suggesting greater immune selection against cells transcribing elongated and multiply spliced HIV transcripts. The larger fold decrease in multiply spliced HIV transcripts accords with findings from our prior study of acute-treated PWH (24).

In the chronic-treated PWH, we also observed decreases from T1 to T2 in the ratios of elongated/initiated HIV RNA and completed/elongated HIV RNA. These findings, which were also observed in acute-treated PWH (24), suggest decreases in HIV transcriptional elongation and completion (respectively) and/or increased clearance of infected cells transcribing elongated and completed HIV transcripts. After normalization of each HIV transcript to levels of the corresponding HIV DNA region at the same time point, we observed a decrease in completed HIV RNA per provirus from T1 to T2 but no significant change in levels of elongated HIV RNA per provirus. These results, which also accord with findings from acute-treated PWH (24), suggest that the decrease in elongated but not completed HIV RNA may have been driven by decreases in HIV DNA, and that different immune mechanisms act to decrease HIV transcriptional completion and/or preferentially clear infected cells producing completed HIV RNA. There may be greater immune selection against cells with completed HIV RNA due to its higher translation potential (more protein coding regions, plus the presence of a polyA tail that enhances stability, nuclear export, and translation), and full-length HIV RNA has been reported to be a ligand for RIG-I (33).

In this study of chronic-treated PWH, we also observed a trend toward an increase in initiated HIV transcripts per provirus from T1 to T2, which seems paradoxical but accords with the findings from acute-treated PWH, in whom the increase was statistically significant (24). Given the very low ratio of elongated/initiated HIV transcripts at both time points, the vast majority of these initiated HIV transcripts are likely to be very short, prematurely terminated transcripts containing only the TAR loop or 5′ LTR. It is possible that these short TAR transcripts are immunologically silent or even confer a survival advantage to infected cells, perhaps by competitively inhibiting proteins that are necessary for elongation of nascent HIV transcripts (34) and/or inhibiting signaling through pattern recognition receptors (35).

After suppression of viremia at T2 (~1 year) and clearance of most productively infected cells, multiply spliced HIV RNA continued to decline from T2 to T3 (~3 years) in the chronic-treated PWH. Although the higher levels of the other HIV transcripts at T2 should have made it easier to detect a decrease, no significant decrease was observed in any other HIV transcript between T2 and T3. The sustained decrease in multiply spliced HIV RNA, which has not previously been reported, suggests differences in the immune responses against non-productively infected cells transcribing multiply spliced HIV RNA, resulting in either progressive silencing of HIV multiple splicing and/or more prolonged clearance of cells expressing multiply spliced HIV RNA. Multiply spliced HIV RNA has a high potential to be translated into Tat or Rev proteins (see Fig. 1), which may trigger adaptive immune responses directed at Tat or Rev. In addition, these HIV proteins could increase HIV transcription (Tat) or nuclear export of intron-containing HIV RNA (Rev), which can further increase expression of viral products that trigger immune responses.

While the large fold decrease in multiply spliced HIV RNA accords with our findings from the acute-treated PWH, the sustained decrease in multiply spliced HIV RNA contrasts with that study, in which no further decreases were detected between 6 months and 1–2 years after suppressive ART (24). In addition, the sustained decrease in multiply spliced HIV RNA contrasts with several other published studies using different assays, which showed that multiply spliced HIV RNA appeared to decline to undetectable or stable levels by 8 weeks (19), 12 weeks (22), or 1 year on ART (13, 21). Possible explanations could include differences in the study participants (including ART start time, immune responses) or methods (including the assay and duration of follow-up). In particular, the higher pre-ART levels of multiply spliced HIV RNA and the longer duration of follow-up in our study may have increased the ability to detect decreases from T2 to T3.

After T3 (~3 years), we did not detect significant decreases in any HIV transcript. The inability to detect further decreases in Pol, completed, multiply spliced, or intact HIV RNA may have been due to the increasing fraction of participants in whom these transcripts could no longer be detected at T3 (5/10, 5/10, 5/10, and 9/10, respectively) as well as the lower number of participants with samples available from T4 and T5. However, the initiated and 5′ elongated HIV RNA remained detectable in all participants and showed no significant decrease after T2. In accord with prior studies in which unspliced HIV RNA appeared to decline to a stable plateau (11, 13, 20–22), our findings suggest attainment of an equilibrium between the production of certain HIV transcripts (most of which are likely incomplete and have lower potential for translation) and the defense mechanisms that may act to reduce HIV transcription and/or clear cells expressing these HIV transcripts.

Using the intact viral RNA assay (7), we also found evidence for differential clearance of intact and defective HIV transcripts. From T1 to T2, intact and 3′ defective HIV RNA decreased significantly, while we did not detect a decrease in 5′ defective HIV RNA. These findings contrast with those in acute-treated PWH, in whom we observed decreases in all three types of HIV RNA over a shorter time on ART, although we had more power in the acute treatment cohort due to the larger number of study participants (24). In those who initiated ART during chronic infection, the fold decrease in intact HIV RNA was greater than that of 5′ defective HIV RNA, suggesting preferential selection against cells transcribing intact HIV RNA. Also, intact HIV RNA differed from all other HIV transcripts measured here in that the levels became undetectable in all individuals at T4–T5. These findings may be explained by the high potential of intact HIV RNA to be translated into Gag and/or Pol proteins (see Fig. 1), which may trigger adaptive immune responses directed against these HIV proteins, as well as the potential of intact HIV RNA to be packaged into virions. The greater fold decrease in intact HIV RNA was also observed in acute-treated PWH (24).

In this study of chronic ART initiation, we found multiple significant correlations between levels of various proviruses and HIV transcripts before and after ART. The number of correlations was lower than observed in the acute-treated PWH (24), which may be due to the smaller number of individuals in this chronic treatment cohort. At T2 and T3 in the chronic-treated PWH, we observed correlations between levels of initiated, 5′ elongated, and completed HIV RNA and levels of the U3-U5 and TAR HIV DNA regions, perhaps because these proviruses have intact 5′ and/or 3′ LTRs that can support HIV transcriptional initiation and/or polyadenylation. However, none of these HIV RNA or DNA levels correlated with intact HIV RNA, multiply spliced HIV RNA, or intact HIV DNA. At T2, intact HIV RNA correlated only with multiply spliced HIV RNA and intact HIV DNA, while at T3, intact HIV RNA and DNA did not correlate with any other measure. These findings could indicate that most of the U3-U5 and TAR-containing proviruses have defects elsewhere, and that most of the initiated, 5′ elongated, and completed HIV RNA are transcribed from defective proviruses, while multiply spliced and intact HIV RNA are more likely to arise from intact proviruses. However, it is also likely that it was harder to find correlations with intact HIV or multiply spliced HIV RNA due to the lower levels and higher number of undetectable samples, which were all assigned the same value.

Like all studies, our study has limitations that deserve further discussion. Although we found many significant *P* values, the relatively low number of study participants may have limited our power to detect some differences or changes, particularly at T4 and T5. In addition, the available cells and nucleic acid had to be divided across multiple different HIV assays, which may have reduced our ability to detect rare HIV targets. Finally, there was some variation between participants in the timing at which samples were obtained relative to the start of ART (see Fig. 2A), which limits our ability to know the exact timeframe over which changes in HIV DNA or RNA were occurring. While it would have been ideal if all participants had samples from the exact times after the start of ART, it is very difficult to obtain longitudinal clinical samples that include timepoints before ART (these samples are usually not available) as well as 2–4 timepoints spanning up to 6–10 years after the start of ART. Also, it should be noted that for any individual study participant, all HIV targets were measured at the exact time, and all timepoints were sequential. Therefore, it is still possible to apply paired statistical tests to determine whether a given HIV target is decreasing over time within individuals and whether there are differences between the various HIV targets, which were the main objectives of our study. To investigate whether the timing of sampling may have affected the HIV RNA levels within a given timepoint, we assessed for correlations between levels of each HIV transcript at T2 and the time post-ART, and we performed a similar analysis for T3. We found no significant correlations, suggesting that the HIV RNA levels at these timepoints were driven more by other factors, such as the variation between participants in the baseline HIV RNA levels and immune responses. Moreover, we did not detect changes in most HIV transcripts after T2, suggesting that most of the decreases had already occurred by T2. In the future, our collaborators will use mathematical models to analyze the data in ways that can account for variation in the timing of samples and can fit data from all timepoints to various models of decay, including those in which decay may occur over multiple phases.

In this study of individuals who started ART during chronic infection, we found differences between the rates at which various HIV proviruses and transcripts decrease with time on ART. In accord with our prior study of acute-treated PWH (24), we found evidence suggesting greater immune selection against cells with completed, multiply spliced, and intact HIV transcripts, which may reflect the higher potential of these HIV transcripts to be translated into HIV proteins. With the much longer follow-up in this study, we found new evidence for more prolonged decay of certain proviruses (R-U5-pre-Gag HIV DNA declined through T4) and HIV transcripts (multiply spliced HIV RNA declined through T3), indicating that their clearance may be dictated by different or more sustained immune mechanisms. With the longer follow-up of 10 years, we also found that some types of HIV transcripts (such as intact HIV RNA) became undetectable over time, while levels of other HIV transcripts (initiated, 5′ elongated, 3′ defective, 5′ defective) and all proviruses appeared to reach an equilibrium after a certain amount of time on ART. These findings provide further evidence for the limits of both the immune system and long-term ART to reduce expression of certain HIV transcripts, particularly incomplete or defective HIV transcripts that may still contribute to immune activation (6). Our findings also suggest the need for new therapies that can target the remaining proviruses/transcripts and/or induce (or permanently block) expression of more immunogenic completed, multiply spliced, and intact HIV transcripts.

## SUPPLEMENTAL MATERIAL

Supplemental figures jvi.00683-25-s0001.docx Figures S1 to S9.

Table S1 jvi.00683-25-s0002.docx Demographic and clinical characteristics.

Table S2 jvi.00683-25-s0003.xlsx Correlation P values.

ASM does not own the copyrights to Supplemental Material that may be linked to, or accessed through, an article. The authors have granted ASM a non-exclusive, world-wide license to publish the Supplemental Material files. Please contact the corresponding author directly for reuse.
