# Supplementary material for: Differential decreases in various HIV DNA regions and HIV transcripts after ART initiation during chronic infection
Source: J Virol. 2025 Jul 8;99(8):e00683-25. doi: 10.1128/jvi.00683-25 (PMC12363177; doi:10.1128/jvi.00683-25)
Supplement: Supplemental figures — Figures S1 to S9. [file jvi.00683-25-s0001.docx]

**Supplementary Figures:**

**
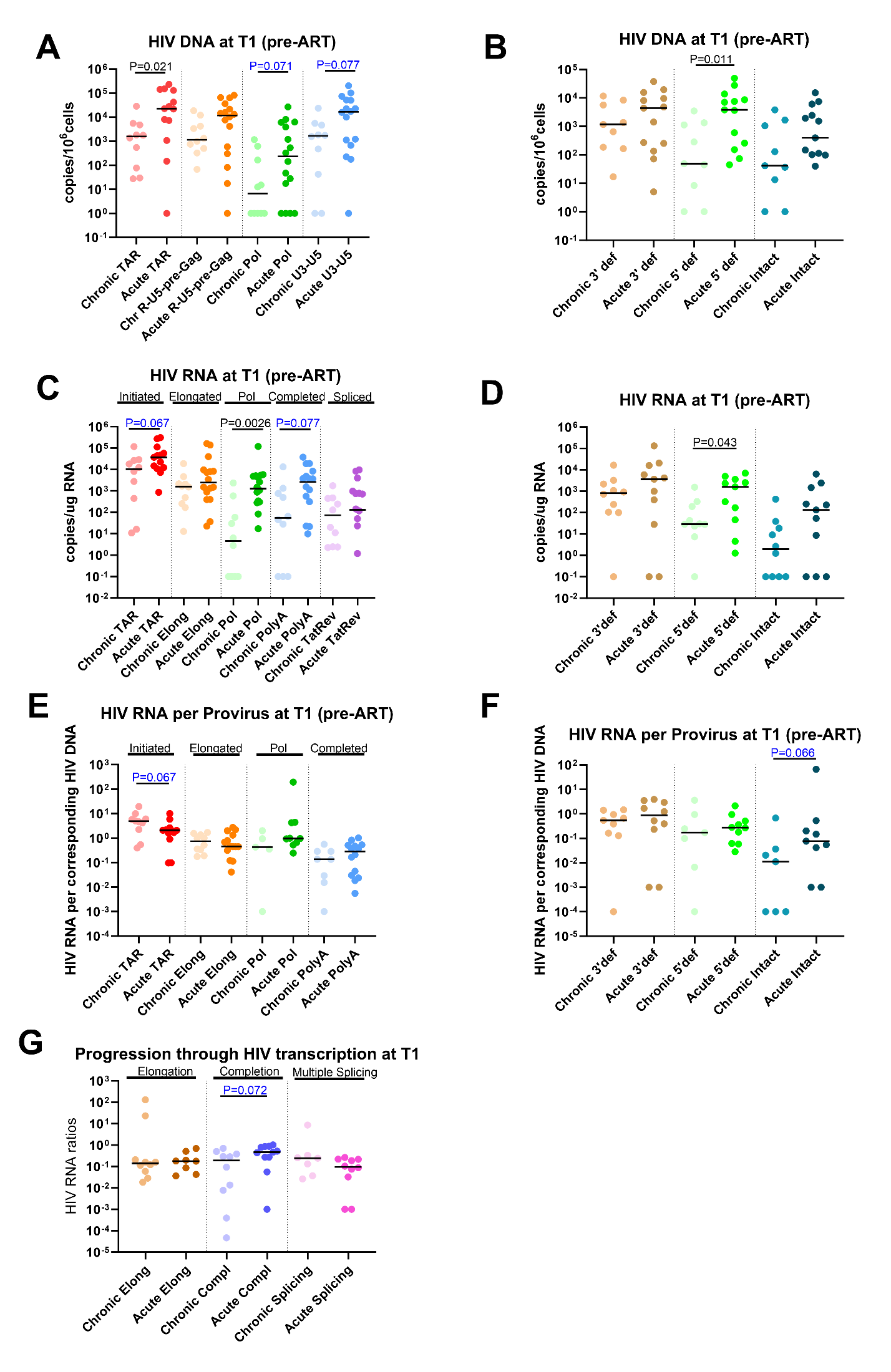
**

**Figure S1: HIV-1 DNA and transcription in untreated chronic vs. acute infection (pre-ART).** HIV DNA and RNA levels were measured in circulating CD4+ T cells prior to the start of ART. A) HIV DNA levels of the TAR, R-U5-pre-Gag, Pol, and U3-U5 regions, as measured by ddPCR and normalized by mass of DNA input; B) Levels of 3’ defective, 5’ defective, and intact proviral DNA, as measured by ddPCR (IPDA) and normalized by DNA input; C) Levels of initiated (TAR), 5’ elongated (R-U5-pre-Gag), Pol (mid transcribed, unspliced), completed (PolyA), and multiply spliced (Tat-Rev) HIV RNA, as measured by RT-ddPCR and normalized by RNA input (copies per 1µg of cellular RNA, which corresponds to about 10^6^ cells); D) Levels of 3’ defective, 5’ defective, and intact HIV RNA, as measured by dd-RT-PCR (IVRA) and normalized by RNA input; E)-F) Levels per provirus of each HIV transcript, as calculated by normalizing the levels of each HIV RNA to levels of the same or corresponding HIV DNA region; G) Progression through the stages of HIV transcriptional elongation, completion, and splicing, as measured by the ratios of elongated/initiated HIV RNA, completed/elongated HIV RNA, and multiply spliced/completed HIV RNA. Bars indicate medians. P-values (two tailed) were calculated using the Mann-Whitney test.

**
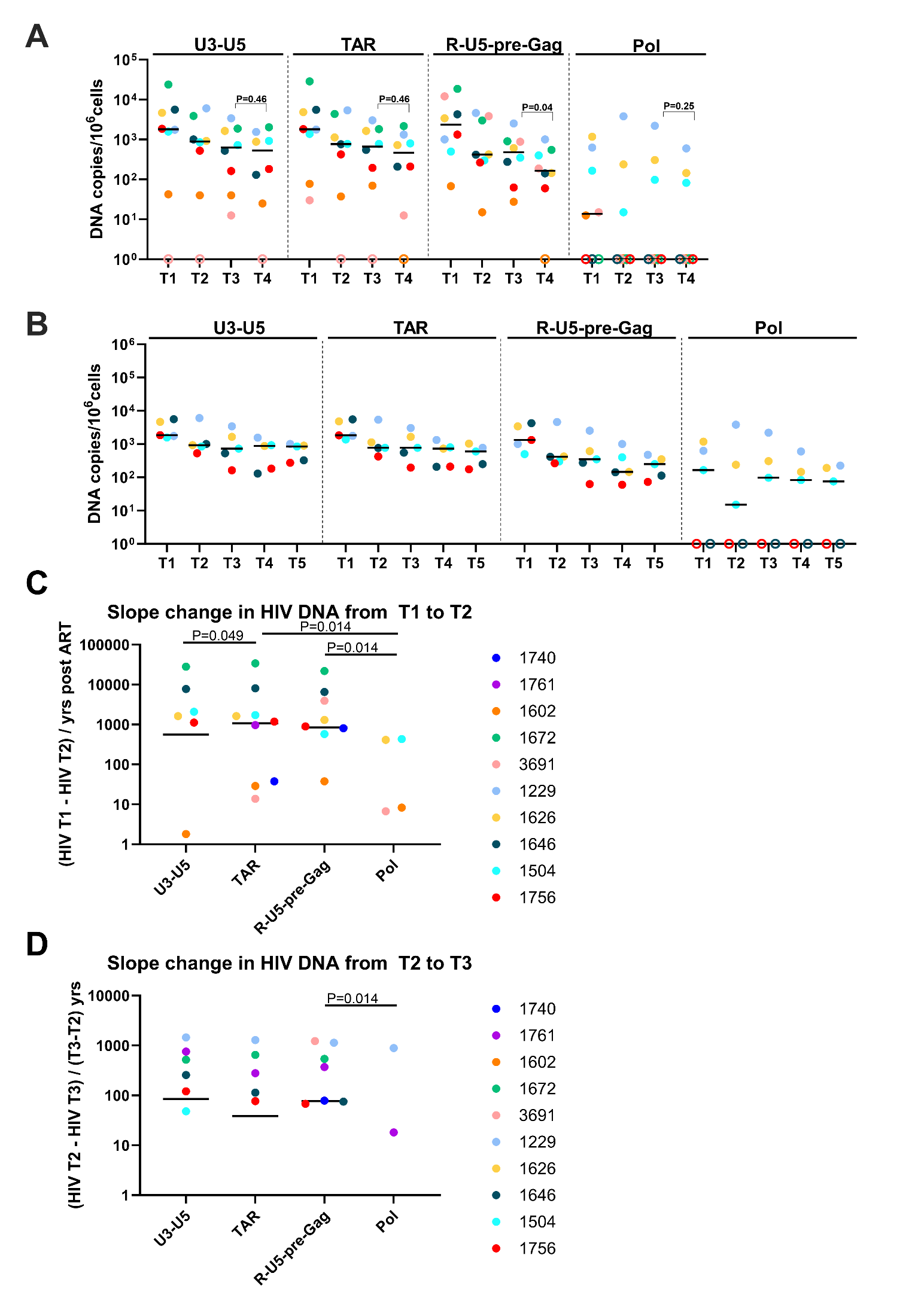
**

**Figure S2: Changes in HIV DNA regions after starting ART.** Levels of U3-U5, TAR, R-U5-pre-Gag, and Pol HIV DNA were measured by ddPCR before ART (T1) and at various times after ART, including: A) T1-T4; and B) T1-T5. For accurate comparison of medians (bars), each graph only shows participants with samples available from all timepoints in that graph. C) Slope change in each HIV DNA region from T1 to T2, as measured by the change in HIV DNA (T1-T2) divided by the time in years between ART start and T2. D) Slope change in each HIV DNA region from T2 to T3, as measured by the change in HIV DNA (T2-T3) divided by the change in time in years (T3-T2). Horizontal lines indicate medians, different colors indicate individual study participants, and open circles indicate undetectable values. P-values (two-tailed) were calculated using the Wilcoxon signed rank test.


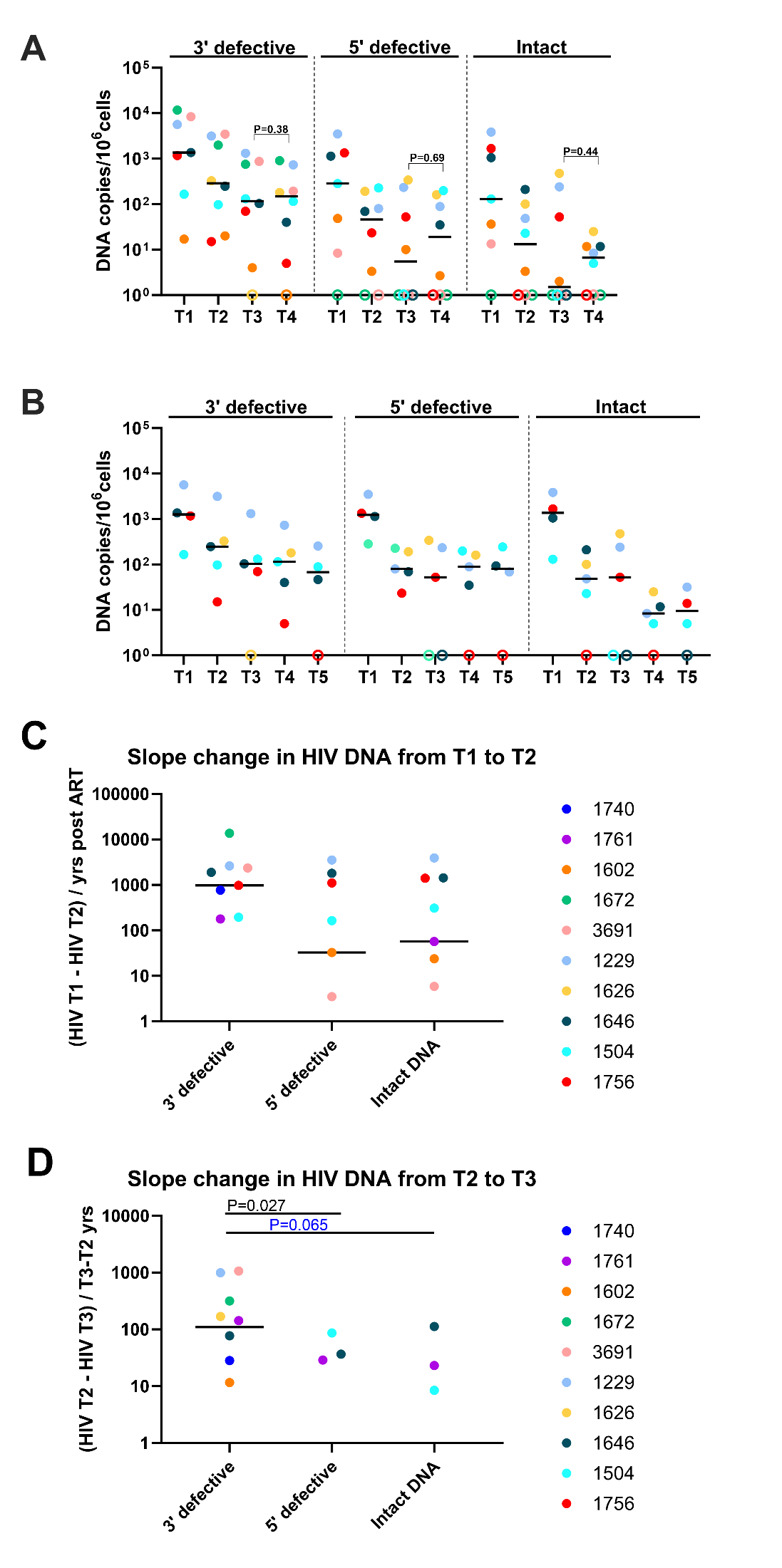


**Figure S3: Change in intact and defective proviruses after ART.** Levels of 3’ defective (Psi+RRE-), 5’ defective (Psi-RRE+) and intact (Psi+RRE+) proviral DNA were measured by ddPCR (IPDA) before ART (T1) and at various times after ART, including: A) T1-T4; and B) T1-T5. For accurate comparison of medians (bars), each graph only shows participants with samples available from all timepoints in that graph. C) Slope change in each provirus from T1 to T2, as measured by the change in HIV DNA (T1-T2) divided by the time in years between ART start and T2. D) Slope change in each provirus from T2 to T3, as measured by the change in HIV DNA (T2-T3) divided by the change in time in years (T3-T2). Horizontal lines indicate medians, different colors indicate individual study participants, and open circles indicate undetectable values. P-values (two-tailed) were calculated using the Wilcoxon signed rank test.


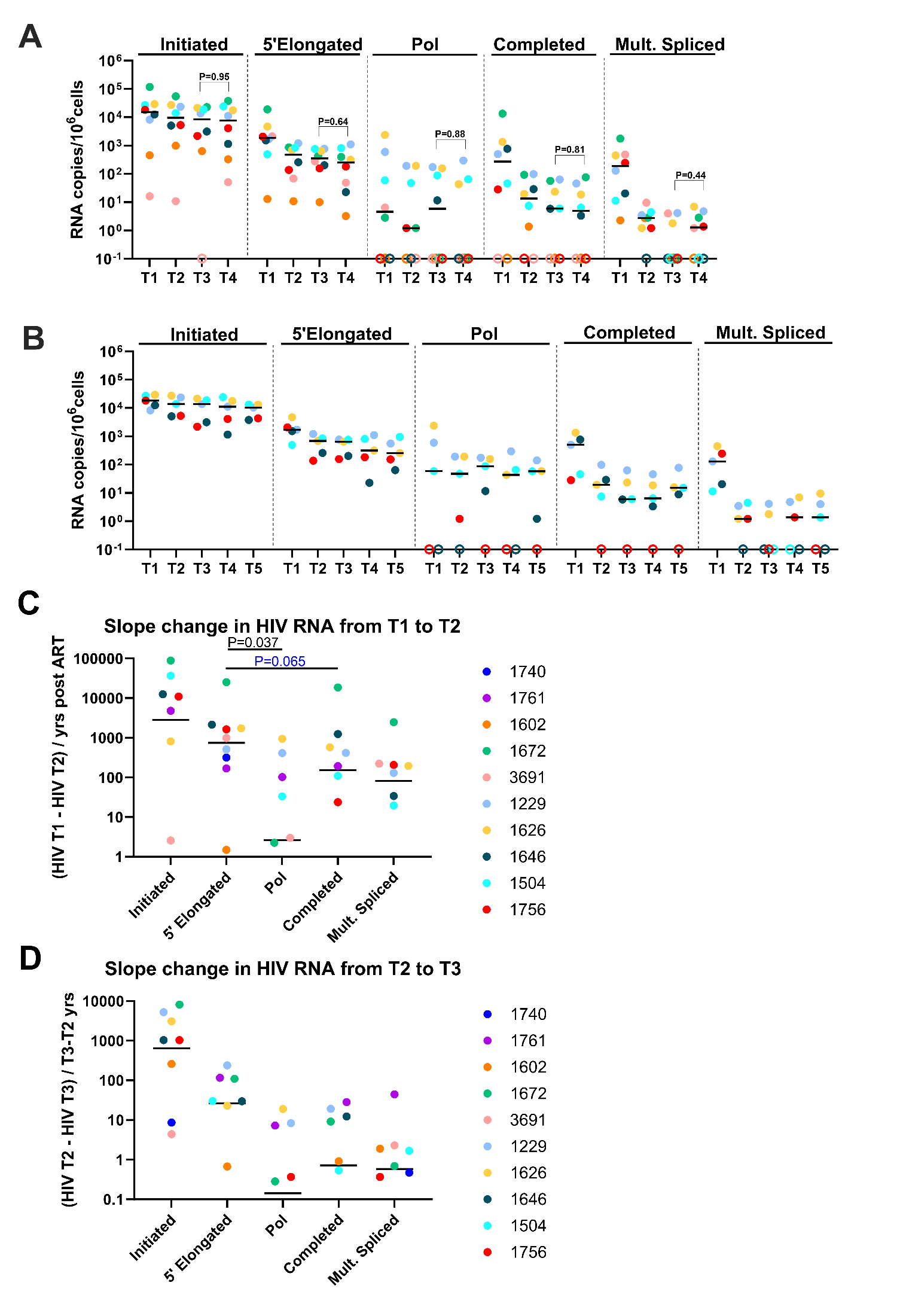


**Figure S4: Changes in levels of different HIV transcripts after ART.** Levels of initiated (TAR), 5’elongated (R-U5-pre-Gag), Pol (mid-transcribed, unspliced), completed (U3-polyA), and multiply spliced (Tat-Rev) HIV RNA were measured by RT-ddPCR before ART (T1) and at various times after ART, including: A) T1-T4; and B) T1-T5. For accurate comparison of medians (bars), each graph only shows participants with samples available from all timepoints in that graph. C) Slope change in each HIV transcript from T1 to T2, as measured by the change in HIV RNA (T1-T2) divided by the time in years between ART start and T2. D) Slope change in each HIV transcript from T2 to T3, as measured by the change in HIV RNA (T2-T3) divided by the change in time in years (T3-T2). Horizontal lines indicate medians, different colors indicate individual study participants, and open circles indicate undetectable values. P-values (two-tailed) were calculated using the Wilcoxon signed rank test.


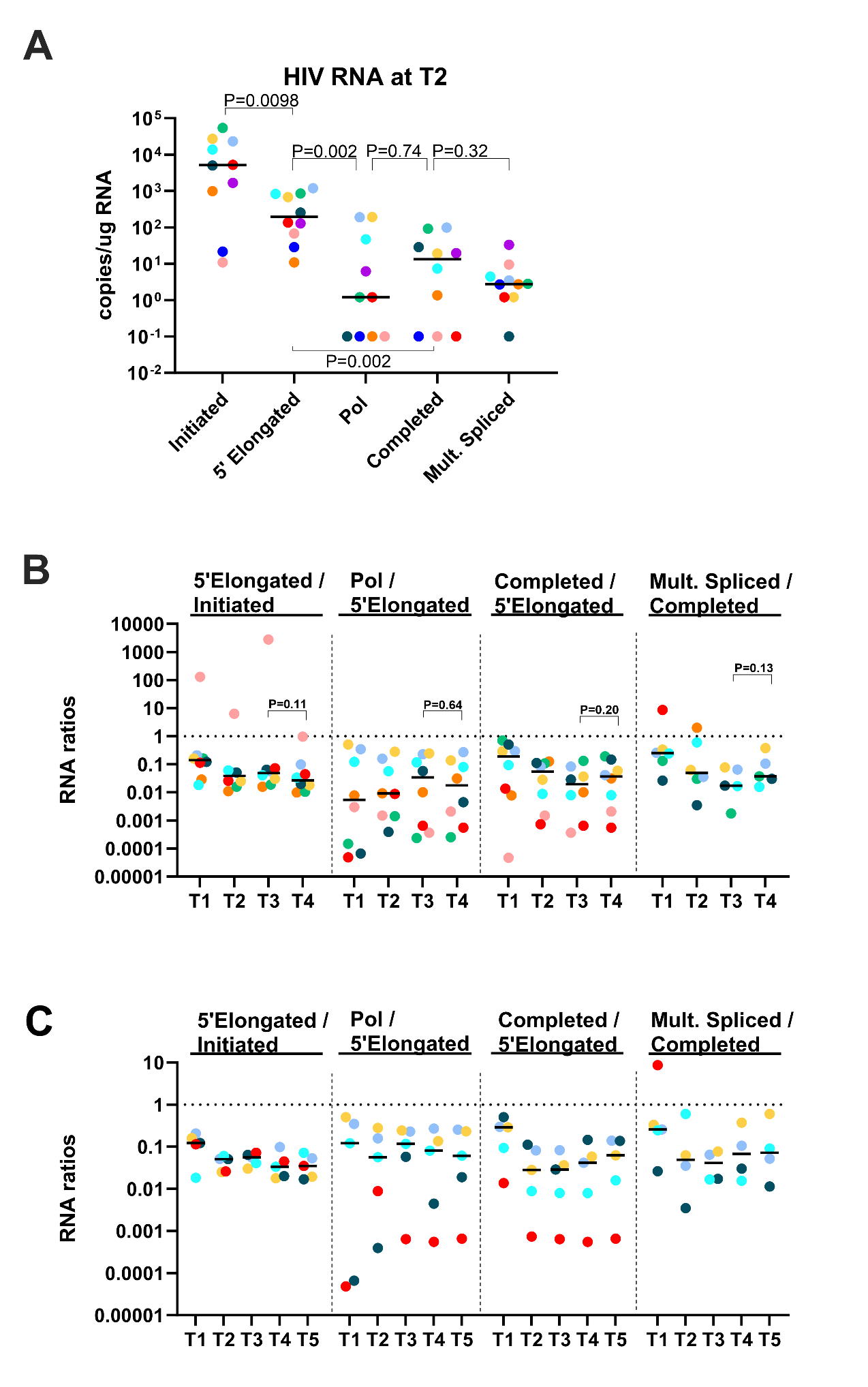


**Figure S5: Changes in progression through stages of HIV transcription after ART.** A) Comparison between levels of different HIV transcripts at T2. B-C) Ratios of one HIV RNA to another, indicating the degree of HIV transcriptional elongation (5’elongated/initiated), mid transcription (Pol/5’elongated), completion (completed/5’elongated), and multiple splicing (multiply spliced/completed) at times T1-T4 (B) or T1-T5 (C). These ratios are independent of infection frequency or progression through prior stages of HIV transcription. For accurate comparison of medians (bars), each graph only shows participants with samples available from all timepoints in that graph. Horizontal lines indicate medians and different colors indicate individual study participants. P-values (two-tailed) were calculated using the Wilcoxon signed rank test.

**
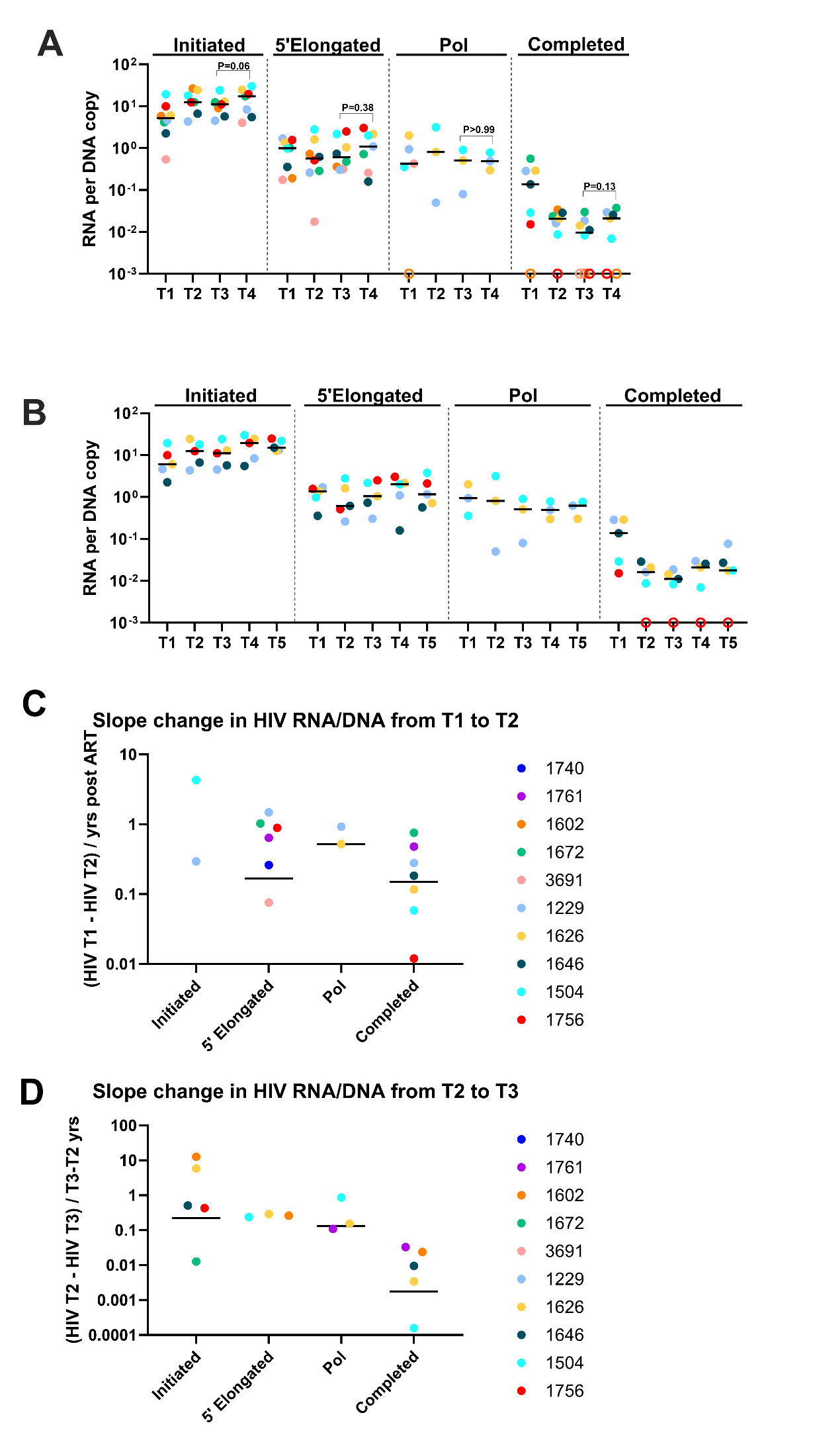
**

**Figure S6: Changes in levels of different HIV transcripts per provirus after ART.** To account for how on-ART decreases in HIV DNA or changes in the relative frequencies of mutations in different HIV DNA regions could affect HIV RNA levels, the levels of each HIV transcript were normalized to levels of the same or corresponding HIV DNA region (HIV RNA/HIV DNA) measured at the same time point. Levels per provirus of initiated (TAR), 5’elongated (R-U5-pre-Gag), Pol (mid-transcribed, unspliced), and completed (U3-polyA) HIV transcripts are shown before ART (T1) and at various times after ART, including: A) T1-T4; and B) T1-T5. For accurate comparison of medians (bars), each graph only shows participants with samples available from all timepoints in that graph. C) Slope change in each HIV transcript per provirus from T1 to T2, as measured by the change in HIV RNA/DNA (T1-T2) divided by the time in years between ART start and T2. D) Slope change in each HIV transcript per provirus from T2 to T3, as measured by the change in HIV RNA/DNA (T2-T3) divided by the change in time in years (T3-T2). Horizontal lines indicate medians, different colors indicate individual study participants, and open circles indicate undetectable values. P-values (two-tailed) were calculated using the Wilcoxon signed rank test.


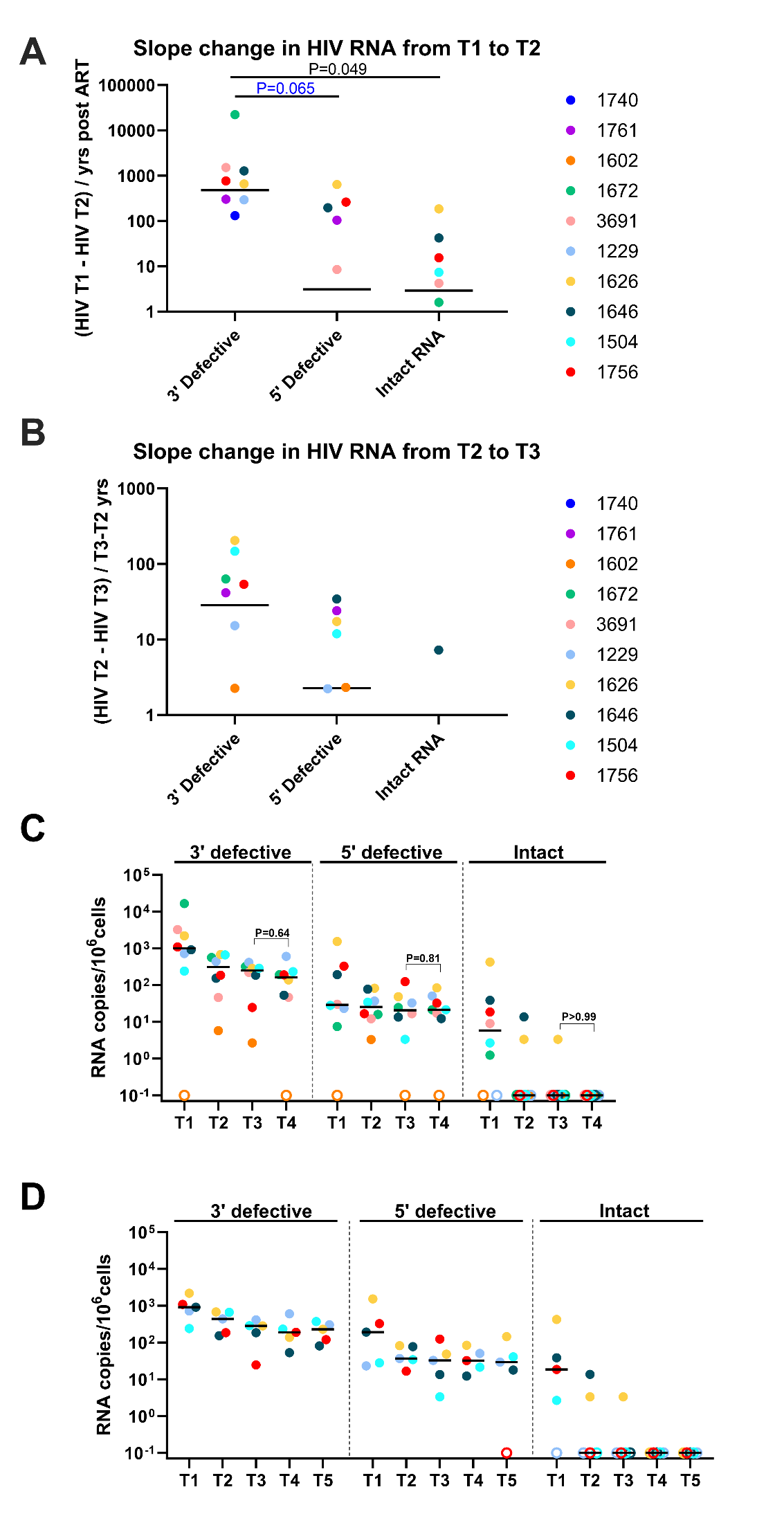


**Figure S7: Changes in intact and defective HIV RNA after ART.** Levels of 3’ defective (Psi+RRE-), 5’ defective (Psi-RRE+) and intact (Psi+RRE+) HIV RNA were measured by dd-RT-PCR (IVDA) before ART (T1) and at various times after ART. A) Slope change in each HIV transcript from T1 to T2, as measured by the change in HIV RNA (T1-T2) divided by the time in years between ART start and T2. B) Slope change in each HIV transcript from T2 to T3, as measured by the change in HIV RNA (T2-T3) divided by the change in time in years (T3-T2). C-D) changes in each HIV transcript from T1-T4 (C) and T1-T5 (D). For accurate comparison of medians (bars), each graph only shows participants with samples available from all timepoints in that graph. Horizontal lines indicate medians, different colors indicate individual study participants, and open circles indicate undetectable values. P-values (two-tailed) were calculated using the Wilcoxon signed rank test.


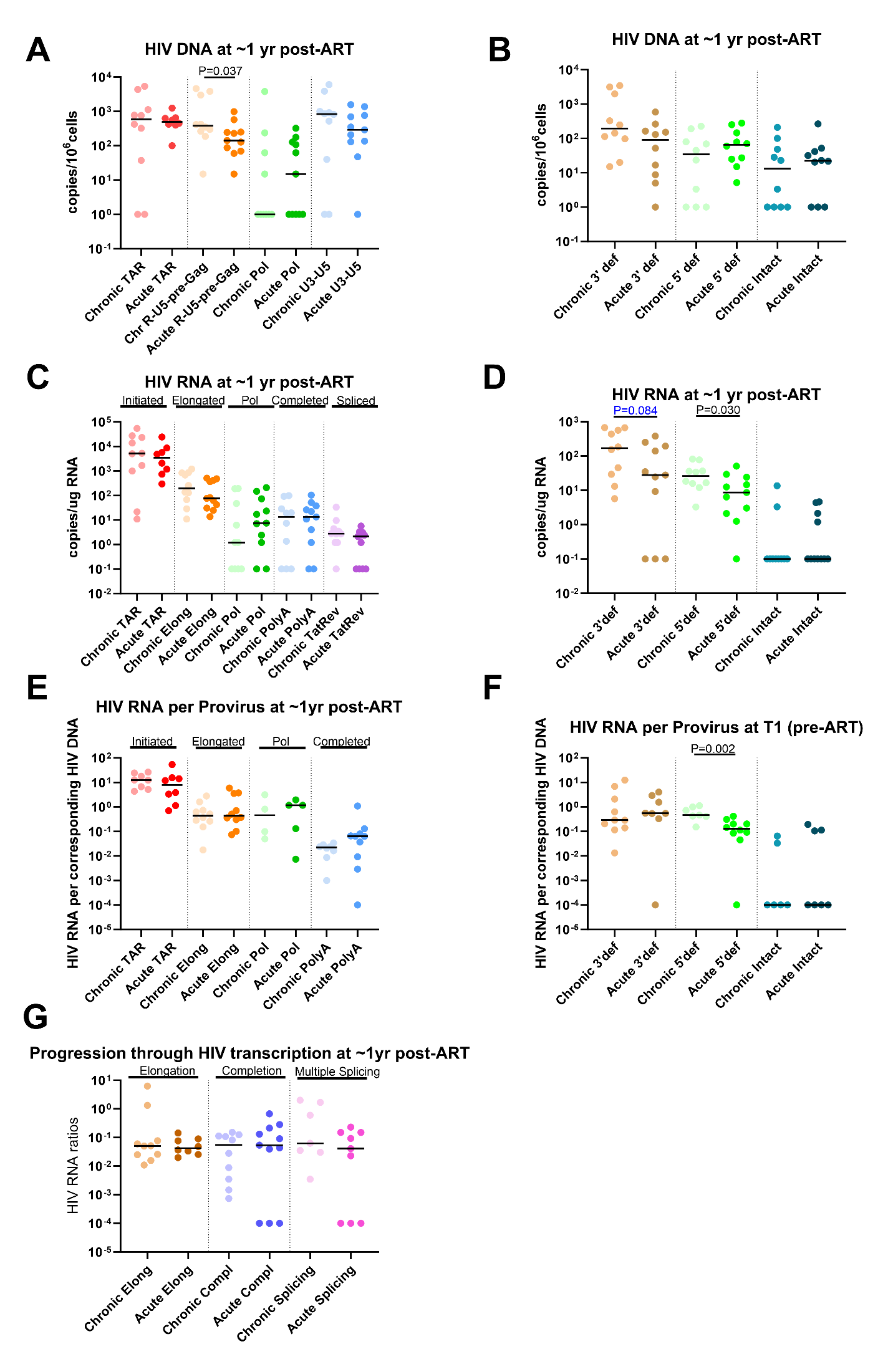


**Figure S8: HIV-1 DNA and transcription after one year of ART in people treated during chronic vs. acute infection.** HIV DNA and RNA levels were measured in circulating CD4+ T cells at one year (T3 from acute treatment study) or approximately one year (T2 from chronic treatment study) after the start of ART. A) HIV DNA levels of the TAR, R-U5-pre-Gag, Pol, and U3-U5 regions, as measured by ddPCR and normalized by mass of DNA input; B) Levels of 3’ defective, 5’ defective, and intact proviruses, as measured by ddPCR (IPDA) and normalized by DNA input; C) Levels of initiated (TAR), 5’ elongated (R-U5-pre-Gag), Pol (mid transcribed, unspliced), completed(PolyA), and multiply spliced (Tat-Rev) HIV RNA, as measured by RT-ddPCR and normalized by RNA input (copies per 1µg of cellular RNA, which corresponds to about 10^6^ cells); D) Levels of 3’ defective, 5’ defective, and intact HIV RNA, as measured by dd-RT-PCR (IVRA) and normalized by RNA input; E)-F) Levels per provirus of each HIV transcript, as calculated by normalizing the levels of each HIV RNA to levels of the same or corresponding HIV DNA region; G) Progression through the stages of HIV transcriptional elongation, completion, and splicing, as measured by the ratios of elongated/initiated HIV RNA, completed/elongated HIV RNA, and multiply spliced/completed HIV RNA. Bars indicate medians. P-values (two tailed) were calculated using the Mann-Whitney test.


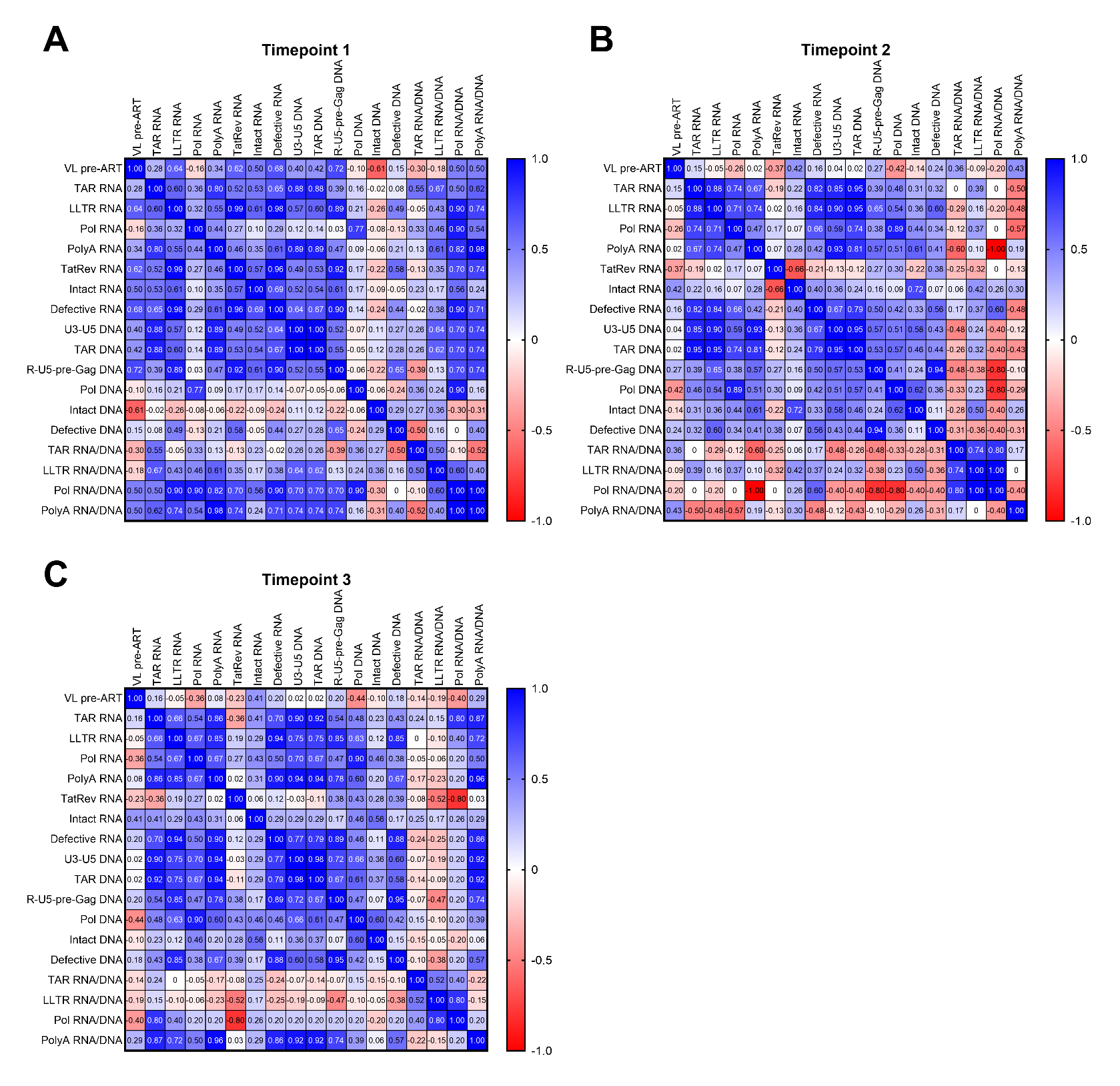


**Figure S9: Spearman correlation analysis between pre-ART viral load (T1) and HIV DNA and RNA measures at T1-T3 in people treated during chronic infection.** Shown are correlation matrices comparing the pre-ART viral load and levels of different HIV transcripts, levels of different HIV DNA regions, and ratios of HIV RNA to the corresponding HIV DNA (average transcription per provirus) at T1 (A), T2 (B), and T3 (C). Numbers indicate r values from Spearman correlations. White numbers indicate significant correlations. Results were not corrected for multiple comparisons. The background of each box has been colored according to r value, as shown in the color scale at right, with darker blue indicating more positive r values and darker red indicating more negative r values. The accompanying P-values are listed in **Table S2**.
