# Supplementary material for: Differential decreases in various HIV DNA regions and HIV transcripts after ART initiation during chronic infection
Source: J Virol. 2025 Jul 8;99(8):e00683-25. doi: 10.1128/jvi.00683-25 (PMC12363177; doi:10.1128/jvi.00683-25)
Supplement: Table S1 — Demographic and clinical characteristics. [file jvi.00683-25-s0002.docx]

**Table S1: Demographic and Clinical Characteristics**

| PID | 1229 | 1504 | 1602 | 1626 | 1646 | 1672 | 1740 | 1756 | 1761 | 3691 | Median |
| --- | --- | --- | --- | --- | --- | --- | --- | --- | --- | --- | --- |
| Age at T1  (yrs) | 38 | 50 | 32 | 45 | 32 | 42 | 48 | 24 | 30 | 29 | 35 |
| Sex | Male | Male | Male | Male | Male | Male | Male | Male | Male | Male |  |
| Race/  ethnicity | White/ European American | Black/ African American | Native American | Hispanic/  Latino | Mixed Ethnicity/Multiracial | White/  European American | Black/  African American | White/ European American | Mixed Ethnicity/ Multiracial | Asian |  |
| Est. time from diagnosis to ART start (yrs) | 6.6 | 25 | 2.1 | 19.8 | 11.1 | 0.8 | 4.7 | 0.5 | 0.5 | 1.1 | 3.4 |
| Maximum recorded viral load | 26209 | 18788 | 40026 | 415301 | 20586 | 302823 | 3368 | 15897 | 2408 | 29328 | 23398 |
| Viral load T1 (copy/ml) | 2192 | 1483 | 9210 | 23000 | 15100 | 235492 | 2763 | 4952 | 2408 | 21972 | 7081 |
| Viral load T2 | <40 | <40 | <40 ND | <40 ND | <40 | <40 | <40 | <40 | <40 | <40 | <40 |
| All VL<40 from T2-T5 | Yes | Yes | Yes | Yes | Yes | Yes | Yes | Yes | Yes | Yes |  |
| CD4 T1  (cells/µl) | 686 | 764 | 736 | 606 | 463 | 414 | 336 | 457 | 971.5 | 362 | 534.5 |
| CD4 T2 | 522 | 550 | 710 | 387 | 472 | 640 | 522 | 668 | 834.5 | 624 | 587 |
| CD4 T3 | 681 | 1338 | 713 | N/A | 845 | 931 | 622 | 767 | 1230.5 | N/A | 806 |
| CD4% T1 | 27 | 20 | 35 | 16 | 25 | 26 | 15 | 27 | 37 | 17.5 | 25.5 |
| CD4% T2 | 21 | 18 | 48 | 20 | 39 | 28 | 30 | 43 | 46 | 30 | 30 |
| CD4% T3 | 27 | 21 | 43.5 | N/A | 36 | 38 | 31 | 27 | 44 | N/A | 33.5 |
| CD8 T1  (cells/µl) | 1058 | 2425 | 608 | 2661 | 1139 | 968 | 1644 | 862 | 1013.5 | 1046 | 1052 |
| CD8 T2 | 1122 | 1782 | 333 | 977 | 577 | 861 | 1049 | 520 | 465.5 | 701 | 781 |
| CD8 T3 | 1007.5 | 2905 | 394.5 | N/A | 993 | 963 | 1037 | 1108 | 908 | N/A | 1000.25 |
| CD8% T1 | 42 | 65 | 29 | 71 | 61 | 61 | 74 | 52 | 39 | 50 | 56.5 |
| CD8% T2 | 46 | 59 | 22 | 51 | 47 | 38 | 59 | 33 | 25.5 | 34 | 42 |
| CD8% T3 | 40.5 | 46 | 24 | N/A | 42 | 39 | 51 | 40 | 33 | N/A | 40.25 |
| ART regimen between T1 and T2 | RGV, FTC/  TDF | 3TC, DRV, RTV, DTG | RPV/  TDF/  FTC | ABC/  DTG/  3TC | EFV/  TDF/  FTC | RAL, FTC/  TDF | ABC/  DTG/  3TC | RPV/  TDF/  FTC, RAL | RAL, FTC/  TDF | DTG, FTC/  TDF |  |
| ART switch | Yes; between T4-T5 | Yes; between T2-T3 | Yes; between T3-T4 | Yes; between T4-T5 | No | Yes; between T2-T3 | No | Yes; between T2-T3 | No | Yes; between T2-T3 |  |
| New ART | DTG,  FTC/  TAF | DTG,  FTC/  TAF | BIC/  FTC/  TAF | CAB,  ABC/  3TC + VRC07 |  | RAL,  ABC/  3TC |  | RPV/  TDF/  FTC,  DTG |  | BIC/  FTC/  TAF |  |
